# Epidemiological and clinical features of Kawasaki disease in Spain over 5 years and risk factors for aneurysm development. (2011-2016): KAWA-RACE study group

**DOI:** 10.1371/journal.pone.0215665

**Published:** 2019-05-20

**Authors:** Elisa Fernandez-Cooke, Ana Barrios Tascón, Judith Sánchez-Manubens, Jordi Antón, Carlos Daniel Grasa Lozano, Javier Aracil Santos, Enrique Villalobos Pinto, Daniel Clemente Garulo, Beatriz Mercader Rodríguez, Matilde Bustillo Alonso, Esmeralda Nuñez Cuadros, Maria Luisa Navarro Gómez, Sara Domínguez-Rodríguez, Cristina Calvo, on behalf of the KAWA-RACE study group

**Affiliations:** 1 Department of Pediatrics, Hospital Universitario Doce de Octubre, Madrid, Spain; 2 Department of Pediatrics, Hospital Universitario Infanta Sofia, Madrid, Spain; 3 Department of Pediatric Rheumatology, Hospital Sant Joan de Deu, Barcelona, Spain; 4 Department of Pediatric Rheumatology, Hospital Parc Tauli, Sabadell, Spain; 5 Department of Pediatrics, Hospital Universitario La Paz, Madrid, Spain; 6 Department of Pediatrics, Hospital Infantil Universitario Niño Jesús, Madrid, Spain; 7 Department of Pediatrics, Hospital Clínico Universitario Virgen de la Arrixaca, Murcia, Spain; 8 Department of Pediatrics, Hospital Universitario Miguel Servet, Zaragoza, Spain; 9 Department of Pediatrics, Hospital Regional Universitario de Malaga, Andalucia, Spain; 10 Department of Pediatrics, Hospital Universitario Gregorio Marañon, Madrid, Spain; 11 Fundación de Investigación Hospital 12 Octubre (Madrid, Spain), Fondazione PENTA ONLUS, Padova, Italy; Careggi University Hospital of Florence, ITALY

## Abstract

**Background:**

Kawasaki disease (KD) is an acute self-limited systemic vasculitis of unknown etiology affecting mainly children less than 5 years of age. Risk factors for cardiac involvement and resistance to treatment are insufficiently studied in non-Japanese children.

**Objective:**

This study aimed to investigate the epidemiology, clinical features and risk factors for resistance to treatment and coronary artery lesions (CAL) in KD in Spain.

**Methods:**

Retrospective study (May 2011-June 2016) of all patients less than 16 years of age diagnosed with KD included in KAWA-RACE network (84 Spanish hospitals).

**Results:**

A total of 625 cases were analyzed, 63% were males, 79% under 5 year-olds and 16.8% younger than 12 months. On echocardiographic examination CAL were the most frequent findings (23%) being ectasia the most common (12%). Coronary aneurysms were diagnosed in 9.6%, reaching 20% in infants under 12 months (*p*<0.001). A total of 97% of the patients received intravenous immunoglobulin (IVIG) with a median number of days from fever onset to IVIG administration of 7.2. A second dose was given to 15.7% and steroids to 14.5% patients. Only 1.4% patients received infliximab. No deaths were reported. A multivariate analysis identified anemia, hypoalbuminemia, hyponatremia, higher creatinine and procalcitonin as independent risk factors for treatment failure and length under 103 cm, hemoglobin < 10.2 mg/dL, platelets > 900,000 cells/mm^3^, maximum temperature < 39.5°C, total duration of fever > 10 days and fever before treatment ≥ 8 days as independent risk factors for developing coronary aneurysms.

**Conclusions:**

In our population, children under 12 months develop coronary aneurysms more frequently and children with KD with anemia and leukocytosis have high risk of cardiac involvement. Adding steroids early should be considered in those patients, especially if the treatment is not started before 8 days of fever. A score applicable to non-Japanese children able to predict the risk of aneurysm development and IVIG resistance is necessary.

## Introduction

Kawasaki disease (KD) is an acute self-limited systemic vasculitis of unknown etiology presenting predominantly in children less than 5 years of age. Diagnosis is based on clinical criteria that include fever, exanthema, conjunctivitis, changes in the extremities, erythema of oral mucosa and lips and cervical lymphadenopathy. The prognosis depends mainly on the extent of cardiac involvement that can be minimized if treatment with intravenous immunoglobulin (IVIG) is administered before the 10^th^ day of disease[1,2].

The etiology of KD is still unknown, although clinical, laboratory and epidemiological features suggest an infectious origin or trigger. However, many studies have failed to identify a unique etiological infectious agent and studies on other biomarkers have tried to recognize the disease early[3,4]. On the other hand, activation of the immune system is characteristic of KD and genomewide association studies have identified a number of plausible loci involved in KD[5–8]. To date, KD diagnosis is still mainly based on clinical criteria although a recent study has described a 13-transcript blood gene expression signature that distinguished KD from other febrile conditions[9].

KD has a universal distribution but is more prevalent in Asian countries, especially in Japan, with an increasing annual incidence of 308 per 100,000 children <5 years in 2014[10]. In Europe incidences range between 4.9 and 15.2 per 100,000 children <5 years of age[11,12]. In Spain, the first population-based study on the epidemiology of KD was published by the KD Catalonian Working Group reporting a mean annual incidence in Catalonia of 3.5/100,000 children <14 years old and 8/100,000 children <5 years (2004–2013)[13]. More recently an incidence of 11.7/100,000 children <5 years has been reported (2005–2015) for the whole of Spain, but this study was a retrospective ecological study and may be overestimating the real incidence[14,15]. Surveillance KD studies are important to monitor trends and outcomes of the disease.

In 2015 a KD study group named KAWA-RACE was set up aiming to investigate the epidemiology, clinical features, risk factors for coronary artery lesions (CAL), specially coronary aneurysms, management and outcome of KD in Spain.

## Materials and methods

Network setup: During 2015 a call for pediatricians who work with KD patients was sent out through the national societies of infectious diseases, rheumatology and cardiology to set up a KD study group that was named KAWA-RACE. A total of 187 pediatricians from 84 Spanish hospitals joined the network. The ethics committee at Instituto de Investigación Hospital 12 de Octubre approved this study (CEIC 15/316). All patient data were fully anonymized before we accessed them.

Data source and collection: We identified all children who were discharged from these hospitals with an International Classification of Diseases (ICD) coding for KD (ICD9 446.1). Retrospective medical data retrieval from 5 years (May 2011–June 2016) was collected and reviewed. A research electronic database capture [16] was created and sent to the participant clinicians together with the study protocol. The patient’s demographic, clinical, laboratory and echocardiographic data were recorded. Egami[17], Sano[18] and Kobayashi[19] scores were calculated.

Subjects and Case Definitions: Individual patient data were reviewed to confirm the diagnosis of KD according to the American Academy of Pediatrics and the American Heart Association (AHA) in 2004[20], including coronary aneurysms criteria. Diagnosis of incomplete KD was made in cases with fewer classical diagnostic criteria and with several compatible clinical, laboratory, or echocardiographic findings, excluding those of other febrile illnesses according to AHA. All patients less than 16 years of age diagnosed with KD diagnosis were included in the study. Exclusion criteria were: Patients older than 16 years at the time of diagnosis, those patients found to be duplicated in the database and patients with a final alternative diagnosis.

Resistance to treatment was defined as persistence of fever 36 hours after the end of IVIG infusion. Minimum follow up was considered 6 months.

Statistical Analysis: Baseline characteristics were described through summary tables reporting frequencies and total records in case of categorical variables and mean (standard deviation) or median (interquartile range (IQR)) when continuous. Chi-squared and Fisher-test (low cell sizes <20) were applied to assess differences among groups for categorical variables. For continuous variables Student T-test and U-Mann-Whitney were applied in parametric and non-parametric distributions respectively. Normality was tested by means of Shapiro-Wilk test. Optimal cutpoint for continuous variables were assessed by means of the maximum AUC values predicting aneurism including 100 bootstrapped samples implemented in cutpoint package[21]. In order to estimate the effect of the different sociodemographic and clinical characteristics with the primary outcomes odds ratios (ORs) were calculated by means of normal and multivariable logistic regression adjusting for age at hospitalization. To test possible non-linear associations with the risk of developing aneurisms, a generalized additive logistic model was performed, and the probability risk of developing aneurism was plotted either for univariate or multivariate analysis. Smoothing parameters defining degrees of freedom (df) were selected according to nested AIC comparisons. Plots were built with ggplot2 and itsadug R packages. R software was used for all analysis[22].

## Results

### Epidemiology

**Fig 1:**
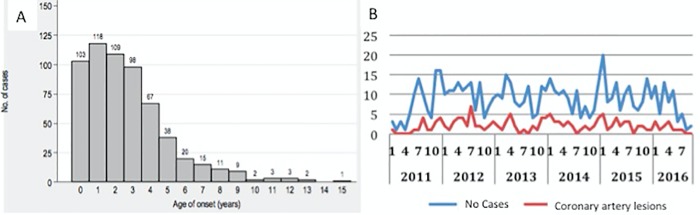
Age (A) and seasonal (B) distribution of Kawasaki disease patients in Spain, from 2011 through 2016.

Nationwide, 654 cases were collected, 29 cases were excluded for not meeting inclusion criteria. Thus, 625 cases were included for analysis. Sixty-three percent were males giving a gender ratio of 1.58:1 and age at diagnosis ranged from 49 days to 15.6 years (median: 2.8 years). Over all 494 (79%) cases occurred in children under 5 years. Forty-two cases (6.7%) were younger than 6 months and 103 cases (16.5%) younger than 12 months. The patients were mainly of European origin (76%) followed by Central and South America (6%) and Asia (5%). A total of 441 were classified as complete (70.5%) and 184 as incomplete (29.5%) KD. Amongst patients younger than 6 and 12 months the proportion of incomplete cases was significantly higher than the group older than 6 and 12 months (52% vs 28%, *p*<0.001) and (25% vs 14%, *p*<0.001), respectively. In 13 (2%) cases it was not the first episode of KD. The onset of KD was more common in winter (28%) and spring (28%), whereas it was relatively less frequent in autumn (19%). Fig 1 represents age (A) and seasonal distribution (B).

Data on family history for first and second degree relatives was available for 560/625 patients and only 5 (0.9%) patients reported relatives with previous KD; in 2 cases it was the mother; in 2 cases, a cousin and in 1 case, a sibling.

### Clinical characteristics

Clinical data of the patients are shown in Table 1. The most common manifestation was fever persisting for 5 days or longer (99.5%) followed by lips and oral changes in both complete and incomplete forms. Only 8% reported that fever was preceded by another symptom. We compared the data between complete and incomplete KD (Table 1) and changes in extremities and acute lymphadenopathy were the least common classical diagnostic criteria present in incomplete cases. Desquamating rash on the groin was significantly less frequent in incomplete KD (17% vs 12%, *p* 0.008).

**Table 1:** Epidemiological and clinical findings in children with Kawasaki disease. Comparison between complete and incomplete cases.

Variable	Complete KD N = 441 (70.5%)	Incomplete KD N = 184 (29.4%)	p-value
**Gender**			
Male	281 (64)	113 (61.4)	0.602
**Age**			
<12 months	60 (13.6)	45 (24.4)	**0.001**
Age at admission (mean), years	2.92 (1.36–4.35)	1.94 (0.86–3.78)	**0.001**
**Classical criteria**			
Conjunctival injection	407 (93.8)	122 (67.0)	**<0.001**
Lips and oral changes	417 (96.3)	146 (79.8)	**<0.001**
Changes in extremities	347 (81.5)	96 (53.3)	**<0.001**
Polymorphous exanthema	403 (94.4)	124 (68.9)	**<0.001**
Acute lymphadenopathy	317 (74.2)	84 (46.1)	**<0.001**
**Other findings**			
Respiratory			
Wheezing	9 (2.0)	7 (3.8)	0.373
Musculoskeletal			
Arthritis, arthralgia (pleocytosis of synovial fluid)	62 (14.0)	21 (11.6)	0.603
Gastrointestinal			
Diarrhea, vomiting, abdominal pain	154 (34.9)	71 (38.8)	0.826
Hepatitis, jaundice	133 (30.1)	60 (33.0)	0.512
Gallbladder hydrops	15 (3.4)	7 (3.8)	0.079
Pancreatitis	1 (0.2)	1 (0.5)	0.827
Nervous system			
Irritability	184 (41.7)	90 (48.9)	0.360
Aseptic meningitis (before IVIG)	8 (1.8)	5 (2.7)	0.097
Sensorineural hearing loss	1 (0.2)	0 (0)	0.626
Genitourinary			
Hematuria	21 (4.7)	11 (6)	0.836
Sterile pyuria	108 (24.5)	54 (32.7)	0.614
Other			
Desquamating rash in groin	74 (17.2)	22 (12.1)	**0.009**

Despite diagnosing and treating for KD, clinicians suspected a different entity in 21% of cases, mainly scarlet fever followed by viral infections. A positive microbiological finding was found in 101 (16%) patients, and they were mainly respiratory viruses (35 cases) and pharyngeal *Streptococcus* group A (29 cases).

On laboratory findings, children with incomplete KD had significantly lower hemoglobin levels (median value 10.6g/L vs 11.0 g/L, *p* <0.001) and higher white cell count (median value 19,500x10^9^/L vs 15,850x10^9^/L, *p* <0.001) (Table 2). The overall terminal segment of the natriuretic atrial peptide (NT-proBNP) before treatment was (median IQR; 394 (180–1034) pg / ml.

**Table 2:** Blood test results in children with Kawasaki disease (KD). Comparison between complete and incomplete cases.

**Variable Median [IQR] n/N**	**Complete KD [N = 441 (70.5%)]**	**Incomplete KD [N = 184 (29.4%)]**	**p-value**
**Hemoglobin g/dl (min)**	11.00 [10.2–11.9] (n = 432)	10.6 [9.8–11.6] (n = 180)	**<0.001**
**White cell count x10**^**9**^**/L (max)**	15,815 [12,650–20,477](n = 350)	19,500 [14,950–22,700] (n = 135)	**<0.001**
**Platelets x10**^**12**^**/L (min)**	327,000 [252,500–402,000] (n = 423)	335,500 [255,250–414,500] (n = 180)	0.883
**Platelets x10**^**12**^**/L (max)**	574,000 [453,500–731,000] (n = 423)	600,500 [458,000–805,500] (n = 180)	0.254
**ESR mm/h**	72.0 [48.0–102.7] (n = 350)	75.0 [43.7–104.7] (n = 140)	0.791
**CRP mg/l**	15.0 [6.9–32.3] (n = 430)	13.2 [7.1–23.3] (n = 180)	0.267
**Albumin g/L**	3.1 [2.7–3.6] (n = 334)	3.2 [2.6–3.8] (n = 146)	0.913
**ALT IU/L**	45.5 [20.0–100.2] (n = 400)	35.0 [20.0–86.5] (n = 170)	0.473
**NT-proBNP pg/ml**	460.5 [200.2–1336.5] (n = 68)	444.0 [228.0–986.5] (n = 18)	0.983

IQR: interquartile range, ESR: Erythrocyte sedimentation rate, CRP: C-reactive protein, ALT: Alanine transaminase, NT-proBNP: N-terminal pro b-type natriuretic peptide

### Heart lesions

Electrocardiographic studies during admission revealed alterations in 47/608 (7.7%) patients, mainly of low voltage in 18 cases, and followed by repolarization alterations in 11 cases.

Echocardiographic examinations were performed during the acute phase of KD in 99% of the cases, among which 32% were found to have an abnormal echocardiogram. CAL were the most frequent findings in 144 patients (23% of cases). Of these, ectasia was the most common lesion, diagnosed in 75/621 patients (12% of cases). Coronary aneurysms were diagnosed overall in 60/621 patients (9.6%) and in 21/103 (20%) infants under 12 months (*p*<0.001). Twenty-eight aneurysms persisted more than 6–8 weeks (4.5% of the total patients). Five cases were diagnosed with giant aneurysms (0.8%). Other data are shown in Table 3. Only 127/621 patients underwent tests looking for peripheral aneurysms and of these 26 (20%) were diagnosed of a peripheral aneurysm, mainly by doppler ultrasound.

**Table 3:** Cardiological findings in children with Kawasaki disease.

Finding	Total patients = 625
**Coronary artery lesions**	144 patients (23%)
Ectasia	75 patients (12%)
**Coronary aneurysms**	60 patients (9.6%)
Number of aneurysms (mean, range)	1.82 (1–6)
Diameter of aneurysms (mean, range)	4.2 (2.1–12) mm
Giant aneurysms (cases)	5 (0.8%)
Number of affected vessels (mean, range)	1.8 (1–4)
Coronary affected	Left main coronary artery (23/60; 38%)
	Right coronary artery (19/60; 31%)
	Left anterior descending artery (13/60; 21%)
	Left circumflex coronary artery (6/60; 10%)
Persistence aneurysms (> 6–8 weeks)	28 (4.5%)
**Valvular involvement**	57 patients (9.2%)
	Mild and transient mitral valve insufficiency (6.7%)
**Pericardial effusion**	48 patients (7.7%)
**Transient left ventricular systolic dysfunction**	7 patients (1.1%)

### Treatment and outcome

Treatment with IVIG was given to 97% of the patients (606 cases). The median number of days since the first day of fever to IVIG administration was 7.2 (range 0–67) and overall 85% of the children (83% with incomplete and 86% with complete KD) received IVIG within the first 10 days from the onset of the disease. Only 9.5% of patients reported side effects, mainly fever recrudescence during infusion (8 cases; 1.3%) and neck stiffness (6 cases; 1%). The majority of patients received acetyl salicylic acid (543/625, 87%). Among the patients treated with IVIG, 15.7% (98 cases) did not respond and received a second dose and 1.6% (10 cases) required a further dose. Steroids were given to 91 (14.5%) patients, 18 of whom (20%) received them as initial therapy together with IVIG. A higher, non-significant proportion of patients that received steroids were < 6 months (23.8% vs 13.8%, *p* 0.124). Only 9 (1.4%) patients received other immunosuppressive therapies, infliximab in all of them. Blood transfusions for anemia were required in 4 (0.6%) patients. Only one case of neurosensory hearing loss was described. Out of the 30 (5%) patients admitted to the pediatric intensive care unit, a significantly higher proportion of them were younger than 6 months (18% vs 4%, *p* = 0.002). The main cause of admission was suspicion of sepsis (37%) and myocardial dysfunction (17%). Permanent sequelae were reported in 27 (4.3%) patients, mainly due to permanent CAL (giant aneurisms in 2 cases; 7% of patients with sequelae); one patient had myocardial dysfunction, another complete atrioventricular heart block and a third hemophagocytic lymphohistiocytosis. No deaths were reported.

### Risk factors for resistance to treatment

A total of 98 (15.7%) children had IVIG resistance and needed a second dose. A univariate analysis was performed to identify the possible risk factors for resistance to treatment (Table 4). Non-responder patients had a higher proportion of aneurysms (15% vs 8%), lower hemoglobin, albumin and sodium in serum and higher maximum platelet and leucocyte count, higher creatinine, and procalcitonin in serum. The clinical data were similar between responders and non-responders except for the presence of rash that was more frequent in those who had resistance to treatment. When a multivariate analysis was performed, anemia, hypoalbuminemia, hyponatremia and higher creatinine and procalcitonin were independent risk factors for non-response to the initial IVIG (Table 4). Although fever until treatment > 8 days was associated with non-response, it did not remain significant in the multivariate analysis and neither did the Kobayasi score.

**Table 4:** Risk factors for resistance to treatment with IVIG. Univariated and multivariated analysis.

	Univariate Analysis OR [95% CIs]	p-value	Multivariate Analysis* OR [95% CIs]	p-value
**Age at hospitalization (y)**				
≤1	1.5 [0.85;2.5]	0.151	-	-
**Hemoglobin (g/dl)**				
<10.2	**2.5 [1.61;3.94]**	**<0.001**	**2.5 [1.54;4.12]**	**<0.001**
**Sodium (meq/L)**				
<136	**3.5 [2.54;6.45]**	**<0.001**	**2.9 [1.7;5.4]**	**<0.001**
**Creatinine (mg/dl)**				
≥0.4	**2.5 [1.5;4.2]**	**<0.001**	**3.2 [1.8;5.9]**	**<0.001**
**Albumin (g/L)**				
<3.3	**2.9 [1.7;5.1]**	**<0.001**	**3.1 [1.8;5.6]**	**<0.001**
**Procalcitonin (ng/mL)**				
≥5.5	**5.5 [2.1;14.0]**	**0.001**	**9.4 [3.3;27.9]**	**<0.001**
**Aneurysm**				
Yes	**1.95 [1.00;3.59]**	**0.049**	**2.3 [1.1;4.4]**	**0.002**
**Fever duration until treatment (d)**				
≥8	**3.0 [1.7;5.4]**	**0.001**	0.7 [0.42;1.2]	0.251
**Kobayashi**				
≥5 points	**1.9 [1.1;3.38]**	**0.031**	1.5 [0.78;2.9]	0.204
**Echocardiogram alterations**				
Yes	**2.2 [1.44;3.48]**	**<0.001**	1.9 [1.1;3.0]	0.012

* Adjusted by Age at hospitalization.

When risk scores of non-response to the initial IVIG were analyzed, children that needed a second dose of IVIG had more frequently a Kobayasi score ≥ 5. No differences were found in the mean Egami and Sano scores between responders and no responders. The sensitivity and specificity of the scores are detailed in Table 5.

**Table 5:** Scores for the prediction of resistance to treatment with IVIG and its applicability in the Spanish population.

	EGAMI	KOBAYASHI	SANO
	≤4 days of illness (1 p)	Na ≤133 (2 p)	Total bilirrubin ≥0.9 mg/dl (1 p)
	ALT >100 U/L (1 p)	≤4 days of illness (2 p)	AST ≥200 U/L (1 p)
	≤300 x10^9^/L platelets (1p)	ALT≥ 100 U/L (1 p)	CRP≥7 mg/dL (1 p)
	CRP ≥8 mg/dL (1 p)	≤300 x10^9^/L platelets (1 p)	
	Age ≤6 months (2 points)	CRP≥10 mg/dL (1 p)	
		Age≤12 months (1 p)	
		≥80% neutrophils (2 p)	
**High risk**	**≥3 points**	**≥5 points**	**≥2 points**
**Coronary artery lesions**			
Sensitivity (%)	26	25	24.6
Specificity (%)	70	75	79
PPV (%)	52	49	47
NPV (%)	42	52	58
**Coronary aneurysms**			
Sensitivity (%)	24	22	14
Specificity (%)	70	70	64
PPV (%)	38	32	23.5
PNV (%)	54	59	49
**Resistance to IVIG**			
Sensitiviity (%)	34	35.4	25
Specificicity (%)	75	77.4	79
PPV (%)	23	25.6	22.4
NPV (%)	84	84	84.4

ALT, alanine aminotransferase; AST, aspartato aminotransferase; CRP, C reactive protein; IVIG, intravenous immunoglobulin; PPV, predictive positive value; PNV, predictive negative value.

### Risk factors for developing coronary aneurysms

A total of 60 children (9.6%) developed coronary aneurysms. A univariate analysis was also performed to identify risk factors for developing coronary aneurysms (Table 6). Children with coronary aneurysms were younger (mean of 23 vs 34 months), weighed less than 14 kg and had a height under 103 cm more frequently. The group between 6 and 12 months developed aneurysms in a significantly high proportion of cases, up to 23%. Males developed aneurysms more frequently. A higher proportion of children that developed aneurisms had duration of fever for longer than 10 days and oral lesions were less common. Hypotension and shock were also more frequent in children without coronary aneurysms. Regarding blood analysis, anemia; higher value of maximum platelets and leucocytes were present in children with aneurisms.

**Table 6:** Risk factors for developing aneurysms. Univariate and multivariate analysis.

	Univariate Analysis OR [95% CIs]	p-value	Multivariate Analysis* OR [95% CIs]	p-value
**Age at hospitalization (y)**				
≤1	**3.4 [1.97; 6.64]**	**0.002**	-	-
**Gender**				
Male	**2.4 [1.2;5.03]**	**<0.001**	-	-
**Weight (kg)**				
≤ 14	**3.1 [1.5,7.0]**	**0.001**	2.2 [0.85;6.1]	0.111
**Length (cm)**				
≤ 103	**5.3 [1.8,7.0]**	**0.001**	**4.9 [1.3;31.7]**	**0.038**
**BMI**				
> 18.3	0.7 [0.2,2.0]	0.112	0.4 [0.1;1.44]	0.230
**Hemoglobin (g/dl)**				
≤ 10.2	**2.4 [1.10;4.42]**	**<0.001**	**2.2 [1.2;4.1]**	**0.012**
**Platelets (cell/mm**^**3**^**)**				
> 900,000	**4.6 [2.4;8.51]**	**<0.001**	**3.3 [1.54;6.99]**	**0.002**
**Leukocytes (cell/mm**^**3**^**)**				
> 16,800	**2.2 [1.1;8.10]**	**<0.001**	1.5 [0.66;3.53]	0.341
**Maximum Temperature (°C)**				
≤ 39.5	**2.7 [1.15;8.15]**	**<0.001**	**2.8 [1.1;9.70]**	**0.006**
**Total fever duration (d) from the beginning of symptoms**				
> 10	**3.6 [2.0;6.3]**	**<0.001**	**4.8 [2.6;9.3]**	**<0.001**
**Fever duration before treatment (d)**				
≥8	**3.0 [1.7;5.4]**	**<0.001**	**3.9 [2.1;7.5]**	**<0.001**

* Adjusted by age at hospitalization and gender.

None of the analyzed scores (Egami, Sano or Kobayashi), nor the type of KD (complete or incomplete) were associated with higher risk of aneurysms. Children who suffered from a previous infection or those with a microbiological identification presented the same proportion of aneurysms. Patients with aneurysms more frequently received steroids and infliximab.

**Fig 2:**
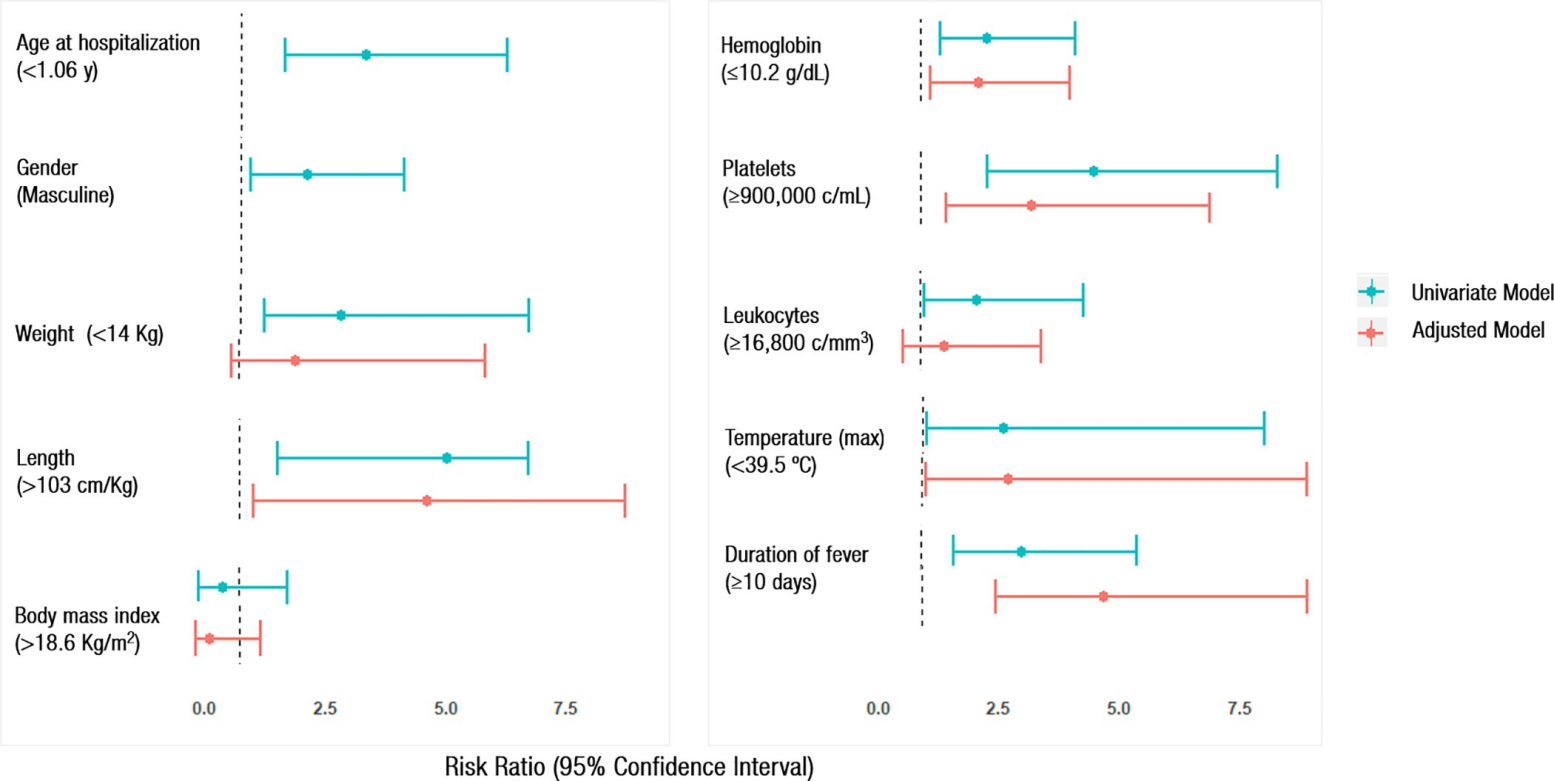
Specific risk factors for developing coronary aneurisms among patients with Kawasaki disease in Spain (2011–2016).

When a multivariated analysis was performed we found that independent risk factors for developing coronary aneurysms were, length under 103 cm; OR = 4.9 [CI 95%: 1.3–31.7], hemoglobin < 10.2 mg/dL; OR = 2.2 [CI 95%: 1.2–4.1], platelets > 900,000 cells/mm^3^; OR = 3.3 [CI 95%: 1.54;6.99], maximum temperature < 39.5°C; OR = 2.8 [CI 95%: 1.1–9.70], duration of fever > 10 days; OR = 4.8 [CI 95%: 2.54–9.11] and fever before treatment ≥ 8 days OR: 3.9 [CI 95%: 2.1;7.5] (Fig 2).

**Fig 3:**
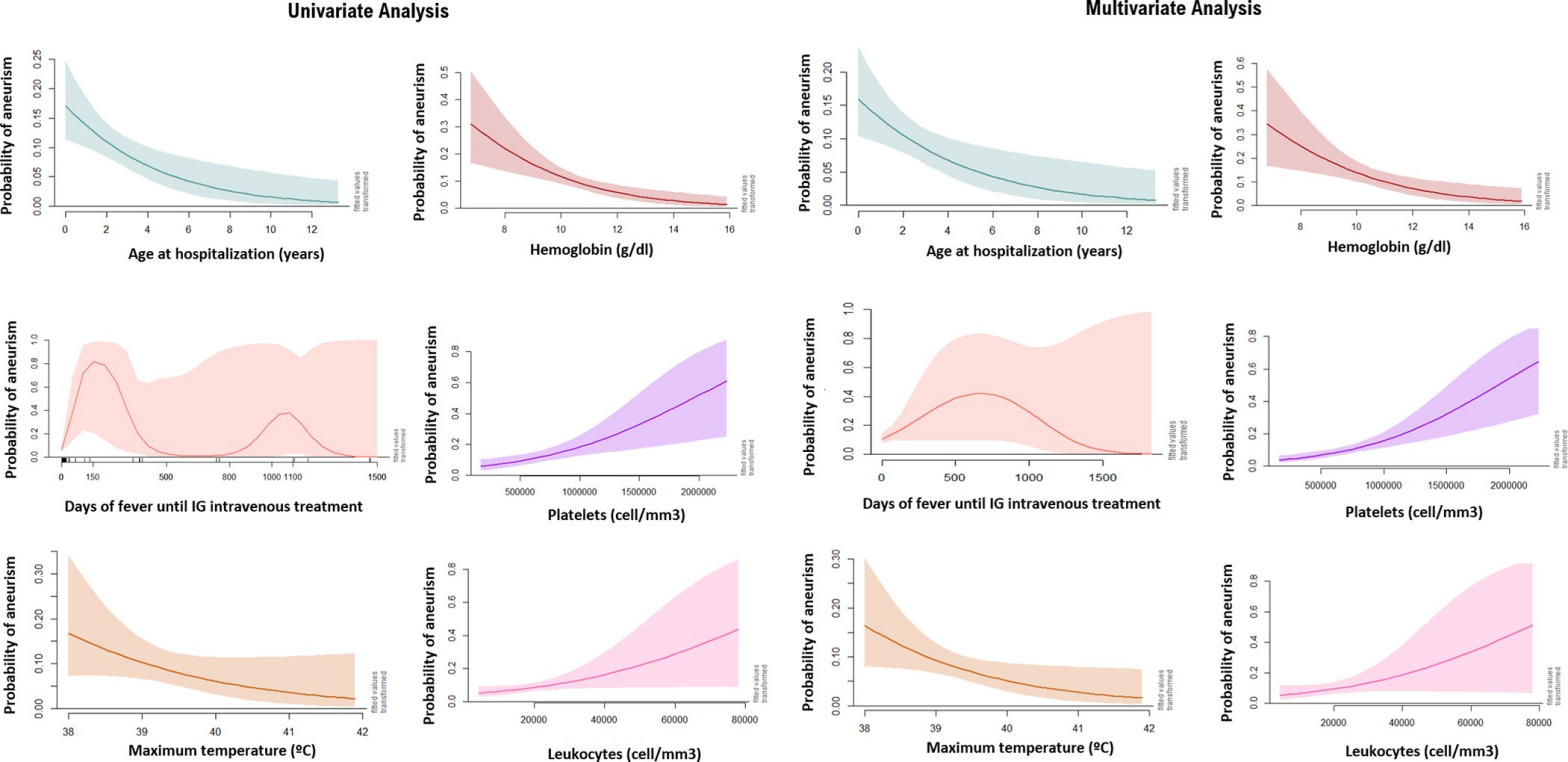
Probability of coronary aneurisms according to specific risk factors among patients with Kawasaki disease in Spain (2011–2016).

According to the generalized additive logistic model, age at hospitalization (df = 4), hemoglobin (df = 5), platelets (df = 6), maximum temperature (df = 5) and leukocytes presented a non-linear association with the probability risk of developing aneurism. Interestingly, days of fever until IVIG treatment (df = 7) presented a concave curve effect in the multivariate and a bimodal for the univariate. The probability of developing aneurisms was higher in younger age at hospitalization (0–4 years), reaching a plateau effect from 8 years of age. A similar association was found for hemoglobin and maximum temperature, when hemoglobin and temperature increase, the probability of aneurisms decreases exponentially until they reach values of 10g/dl and 40°C respectively of plateau effect. Higher levels of platelets and leukocytes increase the risk of developing aneurisms. These effects were more pronounced after 1,000,000 platelets/mm^3^ and 30,000 leukocytes/mm^3^ (Fig 3).

## Discussion

This is the first national multicenter epidemiological and clinical study on KD in Spain. From a total of 625 children with KD, coronary aneurysms were detected in 60 cases (9.6%), and we identified as primary risk factors associated with their development, the presence of anemia; child’s height bellow than 103 cm, maximum platelets greater than 900,000 / mm3, the total duration of fever greater than 10 days and the duration of fever > 8 days before treatment. In our series, resistance to IVIG treatment was found in more than 15% of the cases, with the development of aneurysms being more frequent in those cases. The known predictive scores of treatment failure are not applicable in our population as it has been described in other non-Japanese populations[23,24].

The epidemiology of our patients is similar to that described in other countries around us. Gender and age distribution were similar to most reports, boys were more commonly affected and most of the cases occurred in children <5 years with incidence peaking in children 1 year of age[1,13,25]. The male/female reported ratio is approximately 1.5 to 1 and is consistent with our findings[26]. The most commonly affected race was of European origin representing the main population in our country. The seasonal distribution of incidence rates were maximum during winter and spring as reported in European and most Asian studies [27,28] and differing from some Asian studies that report greater incidence during summer[25].

The clinical manifestations of KD in Spain were very similar to those shown in other studies [13,25,29]. The rate for incomplete forms of KD in our study (29.6%) was within the range of those reported in the literature but lower than the rate described in a Spanish study performed in the region of Catalonia (39.8%) possibly indicating increased awareness of KD in this region[13]. Fever persisting for 5 days or longer was still the most frequent clinical manifestation of KD, followed by changes of lips and the least frequent symptom was acute non-purulent cervical lymphadenopathy[30].

Children with KD typically have leukocytosis. Many children have a normocytic normochromic anemia and platelet counts are usually elevated by the end of the first week of illness with counts exceeding 1 million/mm^3^ in some cases. Inflammatory markers are elevated in nearly all patients with KD. Measurements of the erythrocyte sedimentation rate (ESR) are helpful in assessing the degree of inflammation at diagnosis and before IVIG administration. The analytical values of our patients were also similar to those previously described[30]. In our series, the presence of anemia, hypoalbuminemia, hyponatremia and elevation of procalcitonin were risk factors for non-response to treatment and anemia was also an independent risk factor for development of aneurysms. Chen et al, also found anemia as a risk factor for the development of CAL but did not specify if this also applies to aneurysms alone[25]. Recently, hepcidin has been described as inducing transient hyposideremia, anemia, and hypothesized to influence disease outcomes in KD[31]

The percentage of coronary dilation (ectasia) in our series was lower (12%) than in other series that describe an incidence of up to 30% of children by *Z*-score criteria (*Z*>2), even when treated with high-dose IVIG regimens within the first 10 days of illness[1]. The percentage of coronary aneurysms (9.6%) and giant aneurysms (0.8%) in our cohort is high when compared to Asian series (Japan (1.05 and 0.91%)[10], Korea (1.7 and 0.16%)[32] and Shanghai (1.1 and 0.7%)[25] but smaller than those published in USA (13%) [33], The Netherlands (13.5%)[26], Germany (17%)[28] or United Kingdom (19%)[34] and approximate to those described in series of neighboring countries such as Portugal (8.5%)[35]. This discrepancy may be due to different classification systems and should be interpreted with caution. Mitral regurgitation and pericardial effusion are more frequent in our cases (6.7%, 7.7%) but mild and transient as in other series [29,35]. The disparity in the rate of coronary involvement when compared to Asian series could be related to genetic conditions or to delayed diagnosis of the disease in European countries due to its lower incidence. In Asian countries they treat children earlier than in Europe, on the third or fourth day, due to a high index of suspicion, which can also condition a better prognosis. Compared with the countries in our geographical region, our percentage of aneurysms is low, and could rely on the presence of pediatricians in primary care settings and awareness of the disease. Children with coronary aneurysms received IVIG later than those without (median 10 days vs 7 days) in a study from the UK[34]. In our series, treatment after 8 days was an independent risk factor despite the timing from the first symptom to diagnosis being less than 10 days in 85% of our children.

The efficacy of IVIG administered in the acute phase of KD is well established to reduce the prevalence of CAL with higher doses given in a single infusion having the greatest efficacy[1,36]. In our study treatment with IVIG was given to 97% of the patients, in spite of which, we have a 15% of patients with resistance to treatment, being in these cases more likely the development of aneurysms. This proportion is higher than the 4.9% rate reported by Chen et al[25] but within the range of 10% to 20% described in the recent American Heart Association statement by McCrindle et al in 2017[1]. Steroids were given to 15% of the children in our study; and only a small proportion initially together with IVIG, reflecting that they are used mostly in patients who have a poor response to the initial dose of IVIG. Results from the RAISE (Randomized controlled trial to Assess Immunoglobulin plus Steroid Efficacy for Kawasaki disease) study showed that addition of prednisolone to the standard regimen of IVIG improves coronary artery outcomes in patients with severe KD in Japan[37]. Moreover, a recent Cochrane review concludes that the evidence suggests that treatment with a long course of steroids should be considered for all children diagnosed with KD [38] although there are authors calling to take this with caution in unselected non-Japanese populations as two US trials included in the meta-analysis did not demonstrate significant benefit from primary adjunctive steroids[39]. Therefore, we need to identify patients with high risk of failure to treatment and development of aneurysms in order to achieve an earlier and effective treatment as the validated scores in Asian population (Egami, Sano and Kobayashi) were not of utility in our population. In our series, the development of aneurysms was independently associated with a smaller height of the patients (probably indirectly with age), with anemia < 10.2 gr/dL, with leukocytosis greater than 16,000 cell/mm^3^ and with a treatment delay of more than 8 days. Therefore, when these factors are present, adding steroids to the initial treatment should be considered. Age is considered a risk factor (< 12 months of age), and our results confirmed this, constituting the group of patients that develop aneurysms in a higher proportion. Indeed, we consider that in children younger than 1 year of age, we should be very cautious and consider beginning treatment with steroids together with IVIG, as many authors are recommending. For the same reason, some authors recommend performing an echocardiogram on infants with fever without a source for 7 days or longer with elevated inflammatory markers[1,25]

Our study has several limitations. One of them is that we could not calculate an overall incidence of KD as our network did not achieve total national coverage. The retrospective character of our study is also a main limitation, because of missing data in some parameters. Also, further tests looking for distal lesions in the coronary arteries were made in very few patients so there is a potential underestimation of persistent CAL. Nevertheless, we think that the number of patients collected allow us to draw some conclusions in terms of risk factors, that could be validated in a prospective study already ongoing in the KAWA-RACE group.

More studies are necessary in our population and it would be of interest to validate a score for non-Japanese children to be able to predict the risk of aneurysm development and treatment failure that would allow for selecting patients that would benefit from early steroids or other immunosuppressive treatments. Meanwhile, we recommend being extremely cautious with children under 12 months, with anemia and leukocytosis and to consider adding steroids early in those patients, especially if the treatment is not started before 8 days of fever.

## Supporting information

S1 File KAWA-RACE data set. (XLSX)

S2 File KAWA-RACE code book for data set interpretation. (PDF)
