# Supplementary material for: Epidemiological and clinical features of Kawasaki disease in Spain over 5 years and risk factors for aneurysm development. (2011-2016): KAWA-RACE study group
Source: PLoS One. 2019 May 20;14(5):e0215665. doi: 10.1371/journal.pone.0215665 (PMC6527399; doi:10.1371/journal.pone.0215665)
Supplement: S2 File — (PDF) [file pone.0215665.s002.pdf]

Instituto de Investigacion del Hospital 12 de Octubre

Kawasaki

Data Dictionary Codebook

19/10/2016 10:08

#	Variable / Field Name	Field Label<br>*Field Note*	Field Attributes (Field Type, Validation, Choices, Branching Logic, Calculations, etc.)
Instrument: **Preliminaries**			
1	sujeto_id	ID sujeto	text
2	criterios_diagnosticos	CRITERIOS DIAGNÓSTICOS<br><br>Este apartado es tan sólo de consulta para que todos los investigadores tengamos los mismos criterios y así evitar sesgos<br>Estos son los criterios que se van a utilizar para clasificar los casos de EK en este estudio. No deben confundirse los criterios para clasificación final de los casos de EK con criterios para administrar tratamiento a un paciente con sospecha de Enfermedad de Kawasaki, que deben ser más amplios.<br><br>CRITERIOS DIAGNÓSTICOS PARA ENFERMEDAD DE KAWASAKI COMPLETO<br>(No se tienen que dar todos los criterios a la vez)<br>Definición de caso confirmado<br>Fiebre: ≥ de 5 días (o < 5 días, si resolución de la fiebre coincide con tratamiento para EK)<br>MÁS al menos 4 de los siguientes criterios:<br>1. Exantema: polimorfo, fluctuante, sin vesículas ni costras.<br>2. Inyección conjuntival bilateral, bulbar, no supurativa.<br>3. Alteraciones de labios y mucosa oral: eritema con fisuras en labios, lengua aframbuesada, eritema orofaringeo sin exudados ni ulceraciones. (Basta con una)<br>4. Linfadenopatia ≥ 1.5 cm a menudo unilateral (Un conglomerado adenopático (por palpación o por ecografía) de tamaño superior a 1,5 cm equivale a una adenopatía única de ese tamaño).<br>5. Alteraciones en manos y pies: (Basta con una)<br>• Agudas (eritema de palmas y plantas, edema de manos y pies)<br>• Subagudas (descamación de inicio subungueal, extendiéndose después por las yemas de los dedos y palmas/plantas, sin sobrepasar la muñeca).<br>Se podrá diagnosticar de EK con menos de 4 de los siguientes criterios si se detectan alteraciones coronarias<br><br>CRITERIOS DIAGNÓSTICOS PARA ENFERMEDAD DE KAWASAKI INCOMPLETO<br>(No se tienen que dar todos los criterios a la vez)<br><br>Fiebre ≥5 días y ≥2 criterios clínicos<br>MÁS<br>PCR >30mg/l y/o VSG>40mm/h<br>MÁS<br>≥ 3 de las siguientes alteraciones:<br>• Albúmina ≤3gr/dl<br>• Anemia según edad del niño<br>• Elevación de la ALT<br>• Leucocitosis >15.000/mm3 con neutrofilia<br>• Orina >10cel/campo<br>• Plaquetas > 450.000/mm3 después de 7 días<br><br>CRITERIOS DIAGNÓSTICOS PARA ENFERMEDAD DE KAWASAKI ATÍPICO<br>Este diagnóstico se reserva para aquellos casos de Enfermedad de Kawasaki que se presenta con signos o síntomas que generalmente no se ven en esta enfermedad como :<br>• Alteración de la función renal<br>• Abdomen agudo, etc<br><br>CRITERIOS DIAGNÓSTICOS PARA SÍNDROME HEMOFAGOCÍTICO<br>El diagnostico de síndrome hemofagocitico (SHF) se puede	descriptive

		establecer si se cumplen los criterios (1) o (2) abajo descritos:<br>(1) Diagnóstico molecular compatible con síndrome hemofagocitico.<br>(2) Cumplimiento de los criterios diagnósticos (5 de los 8 abajo descritos).<br>(A) Criterios diagnósticos iniciales (evaluados en todos los pacientes con SHF)<br> 1. Fiebre<br> 2. Esplenomegalia<br> 3. Citopenias (que afectan 2 de 3 series en sangre periférica)<br> Hemoglobina < 90 g/L (en niños < 4 semanas: hemoglobina < 100 g/L)<br> Plaquetas < 100_109/L<br> Neutrófilos < 1.0_109/L<br> 4. Hipertrigliceridemia y/o hipofibrinogenemia:<br> Triglicéridos en ayunas ≥ 3.0 mmol/L (i.e., ≥ 265 mg/dl)<br> Fibrinógeno ≤1.5 g/L<br> 5. Hemofagocitosis en medula ósea o bazo o ganglios.<br> 6. No hay evidencia de malignidad<br>(B) Criterios diagnósticos nuevos<br> 7. Actividad NK-celular baja o ausente (según laboratorio de referencia)<br> 8. Ferritina ≥500 mg/L<br> 9. CD25 Soluble (i.e., receptor IL-2 soluble) ≥2,400 U/ml<br>Comentarios:<br>(1) Si la actividad hemofagocítica no está probada en el momento de presentación se recomienda la búsqueda activa de su actividad.<br>Si la muestra médula ósea no es concluyente se obtendrá muestra de otros órganos. Médulas óseas seriadas en el tiempo pueden ser de ayuda.<br>(2) Los siguientes hallazgos proporcionan fuerte evidencia a favor del diagnóstico: :<br>(a) LCR con pleocitosis (células mononucleares) y/o proteínas elevadas en LCR.<br>(b) Imágenes histológicas del hígado compatibles con hepatitis crónica persistente en la biopsia.<br>(3) Otras anomalías clínicas compatibles con el diagnóstico son: síntomas neurológicos, ganglios linfáticos aumentados, ictericia, edema, exantema, anomalías en enzimas hepáticas, hipoproteinemia, hiponatremia, VLDL elevada, HDL descendida.	
3	preliminaries_complete	Complete?	dropdown<br>0 Incomplete<br>1 Unverified<br>2 Complete
Instrument: **Registro**			
4	tipo_registro	Tipo de registro	radio<br>1 Retrospectivo<br>2 Prospectivo<br>Custom alignment: RH
5	especialidad	Especialidad persona que rellena	radio<br>1 Infecciosas<br>2 Reumatología<br>3 Cardiologia<br>4 Pediatria general<br>5 Otros (especificar)<br>Custom alignment: LV
6	especialidad_otra<br>Show the field ONLY if: [especialidad] = '5'	Especificar	text
7	fecha_nac	Fecha de nacimiento del paciente<br>*(dd/mm/aaaa)*	text (date_dmy)

8	sexo	Sexo del paciente	radio<br>1 Hombre<br>2 Mujer<br><br>Custom alignment: RH
9	comentarios_1	Comentarios	notes<br>Custom alignment: LV
10	registro_9d4b_complete	Complete?	dropdown<br>0 Incomplete<br>1 Unverified<br>2 Complete
Instrument: **Datos sociodemográficos**			
11	ccaa	Comunidad Autónoma de procedencia del paciente	dropdown<br>1 Andalucía<br>2 Aragón<br>3 Asturias<br>4 Baleares<br>5 Canarias<br>6 Cantabria<br>7 Castilla-La Mancha<br>8 Castilla y León<br>9 Cataluña<br>10 Extremadura<br>11 Galicia<br>12 La Rioja<br>13 Comunidad de Madrid<br>14 Murcia<br>15 Navarra<br>16 País Vasco<br>17 Comunidad Valenciana<br>18 Ceuta<br>19 Melilla
12	fecha_ingreso_hospital	Fecha de ingreso en el Hospital de referencia<br>*(dd/mm/aaaa)*	text (date_dmy)
13	pat_trasladado	¿Paciente trasladado de otro hospital?	radio<br>1 Si<br>0 No<br><br>Custom alignment: RH
14	fecha_ingreso<br>Show the field ONLY if: [pat_trasladado] = '1'	Fecha de ingreso en dicho hospital<br>*(dd/mm/aaaa)*	text (date_dmy)
15	reside_poblacion	Población de residencia (municipio)	text<br>Custom alignment: RH
16	etnia_pat	Etnia del Paciente (Elija la opción más correcta del menú desplegable)	radio<br>1 Europa del sur (países mediterráneos y Portugal)<br>2 Norte/este Europa<br>3 Gitano<br>4 Afroamericano<br>5 Afrocaribeño<br>6 África subsahariana<br>7 Norte de África (Marruecos, Argelia, Túnez, Libia, Egipto)<br>8 Asia del Sur (India, Pakistán, Bangladesh, Tamil)<br>9 Sudeste Asiático (Vietnam, Camboya, Tailandia, Malasia, Indonesia, Filipinas)<br>10 Asia Occidental (Afganistán, Irán)<br>11 Asia Oriental (China, Japón, Corea)<br>12 Turco

			13 Península arábica<br>14 Oriente Medio<br>15 Judío<br>16 centro/sur Americano nativo<br>17 Otro/mixto (especificar)<br>99 No disponible<br><br>Custom alignment: LV
17	etnia_pat_otra<br>Show the field ONLY if: [etnia_pat] = '17'	Especificar	text
18	consanguinidad_familiar	¿Existe consanguinidad en la familia?	radio<br>1 Si<br>0 No<br><br>Custom alignment: RH
19	consanguinidad_familiar_espe<br>Show the field ONLY if: [consanguinidad_familiar] = '1'	Espeificar	text
20	etnia_madre	Etnia de la Madre (Elija la opción más correcta del menú desplegable)	radio<br>1 Europa del sur (países mediterráneos y Portugal)<br>2 Norte/este Europa<br>3 Gitano<br>4 Afroamericano<br>5 Afrocaribeño<br>6 África subsahariana<br>7 Norte de África (Marruecos, Argelia, Túnez, Libia, Egipto)<br>8 Asia del Sur (India, Pakistán, Bangladesh, Tamil)<br>9 Sudeste Asiático (Vietnam, Camboya, Tailandia, Malasia, Indonesia, Filipinas)<br>10 Asia Occidental (Afganistán, Irán)<br>11 Asia Oriental (China, Japón, Corea)<br>12 Turco<br>13 Península arábica<br>14 Oriente Medio<br>15 Judío<br>16 centro/sur Americano nativo<br>17 Otro/mixto (especificar)<br>99 No disponible<br><br>Custom alignment: LV
21	etnia_madre_otra<br>Show the field ONLY if: [etnia_madre] = '17'	Especificar	text
22	etnia_padre	Etnia del Padre (Elija la opción más correcta del menú desplegable)	radio<br>1 Europa del sur (países mediterráneos y Portugal)<br>2 Norte/este Europa<br>3 Gitano<br>4 Afroamericano<br>5 Afrocaribeño<br>6 África subsahariana<br>7 Norte de África (Marruecos, Argelia, Túnez, Libia, Egipto)<br>8 Asia del Sur (India, Pakistán, Bangladesh, Tamil)<br>9 Sudeste Asiático (Vietnam, Camboya, Tailandia, Malasia, Indonesia, Filipinas)<br>10 Asia Occidental (Afganistán, Irán)<br>11 Asia Oriental (China, Japón, Corea)<br>12 Turco

			13 Península arábica<br>14 Oriente Medio<br>15 Judío<br>16 centro/sur Americano nativo<br>17 Otro/mixto (especificar)<br>99 No disponible<br><br>Custom alignment: LV
23	etnia_padre_otra<br>Show the field ONLY if: [etni a_padre] = '17'	Especificar	text
24	viaje_fuera	¿Ha viajado en el último mes fuera de España?	radio<br>1 Si<br>0 No<br>99 No disponible<br><br>Custom alignment: RH
25	fecha_viaje<br>Show the field ONLY if: [viaj e_fuera] = '1'	Fecha inicio del viaje<br>*(dd/mm/aaaa)*	text (date_dmy)
26	duracion_viaje<br>Show the field ONLY if: [viaj e_fuera] = '1'	Duración del viaje<br>*días*	text (integer)
27	lugar_viaje<br>Show the field ONLY if: [viaj e_fuera] = '1'	Lugar o País	text
28	fprimera_consulta	Fecha 1ª consulta médica por el episodio<br>*(dd/mm/aaaa)*	text (date_dmy)
29	lugar_consulta	Lugar 1ª consulta por síntomas relacionados con EK	radio<br>1 Centro de atención primaria<br>2 Urgencias<br>3 Consultas externas<br>4 Otros (especificar)<br>99 No disponible<br><br>Custom alignment: LV
30	lugar_consulta_otro<br>Show the field ONLY if: [luga r_consulta] = '4'	Especificar	text
31	fecha_diagnostico	Fecha de sospecha clínica de Enfermedad de Kawasaki<br>*(dd/mm/aaaa)*	text (date_dmy)
32	lugar_diagnostico	Lugar donde se inicia la sospecha clínica de Enfermedad de Kawasaki	radio<br>1 Centro de atención primaria<br>2 Urgencias<br>3 Consultas externas<br>4 Planta hospitalización<br>5 Otros (especificar)<br>99 No disponible<br><br>Custom alignment: LV
33	lugar_diagnostico_otro<br>Show the field ONLY if: [luga r_diagnostico] = '5'	Especificar	text
34	antecedentes_familiares	Antecedentes familiares relevantes al caso	radio<br>1 Si<br>0 No<br>99 No disponible<br><br>Custom alignment: RH
35	parentesco<br>Show the field ONLY if: [ant ecedentes_familiares] = '1'	Parentesco	radio<br>1 Padre<br>2 Madre<br>3 Hermano<br>4 Abuelo/a<br>5 Tío/a

			6 Primo/a<br>7 Otro (especificar)<br>Custom alignment: LV
36	parentesco_hermano<br>Show the field ONLY if: [parentesco] = '3'	Especificar	radio<br>1 No gemelo<br>2 Gemelo no idéntico<br>3 Gemelo idéntico<br>Custom alignment: LV
37	parentesco_otro<br>Show the field ONLY if: [parentesco] = '7'	Especificar	text
38	tipo_enfermedad<br>Show the field ONLY if: [parentesco] <> ''	Tipo de enfermedad	radio<br>1 Enfermedad Kawasaki<br>2 Aterosclerosis<br>3 Enfermedades cardiacas relevantes<br>4 Enfermedades autoinmunes (especificar)<br>5 Inmunodeficiencia (especificar)<br>6 Proceso infeccioso en las ultimas 4 semanas<br>Custom alignment: LV
39	enfer_cardiaca<br>Show the field ONLY if: [tipo_enfermedad] = '3'	Especificar	radio<br>1 Aneurisma coronario<br>2 Infarto<br>3 Cardiopatía isquémica en < 45 años<br>4 Muerte súbita de causa cardiológica en < 45 años<br>5 Historia previa de aneurismas familiares<br>6 Otros (especificar)<br>Custom alignment: LV
40	enf_cardiaca_otra<br>Show the field ONLY if: [enfer_cardiaca] = '6'	Especificar	text
41	enf_autoinmune<br>Show the field ONLY if: [tipo_enfermedad] = '4'	Especificar	text
42	enf_inmunodeficiencia<br>Show the field ONLY if: [tipo_enfermedad] = '5'	Especificar	text
43	infeccion_foco<br>Show the field ONLY if: [tipo_enfermedad] = '6'	Foco	radio<br>1 ORL<br>2 Infección respiratoria de vías bajas<br>3 Neumonía<br>4 Infección del tracto urinario<br>5 Meningitis<br>6 Infección de piel y partes blandas<br>7 Sepsis/bacteriemia<br>8 Otros (especificar)<br>Custom alignment: LV
44	infeccion_foco_otro<br>Show the field ONLY if: [infeccion_foco] = '8'	Especificar	text
45	infeccion_patogeno<br>Show the field ONLY if: [tipo_enfermedad] = '6'	¿Se identificó patógeno?	radio<br>1 Si<br>0 No<br>Custom alignment: RH
46	infeccion_patogeno_espe<br>Show the field ONLY if: [infeccion_patogeno] = '1'	Especificar	text
47	otro_familiar<br>Show the field ONLY if: [antecedentes_familiares] = '1'		checkbox<br>1 otro_familiar___1 Añadir otro familiar
48		Parentesco	radio

	parentesco2<br>Show the field ONLY if: [otro_familiar(1)] = '1'		1 Padre<br>2 Madre<br>3 Hermano<br>4 Abuelo/a<br>5 Tío/a<br>6 Primo/a<br>7 Otro (especificar)<br><br>Custom alignment: LV
49	parentesco_hermano2<br>Show the field ONLY if: [parentesco2] = '3'	Especificar	radio<br>1 No gemelo<br>2 Gemelo no idéntico<br>3 Gemelo idéntico<br><br>Custom alignment: LV
50	parentesco_otro2<br>Show the field ONLY if: [parentesco2] = '7'	Especificar	text
51	tipo_enfermedad2<br>Show the field ONLY if: [parentesco2] <> ''	Tipo de enfermedad	radio<br>1 Enfermedad Kawasaki<br>2 Aterosclerosis<br>3 Enfermedades cardiacas relevantes<br>4 Enfermedades autoinmunes<br>5 Inmunodeficiencia<br>6 Proceso infeccioso en las ultimas 4 semanas<br><br>Custom alignment: LV
52	enfer_cardiaca2<br>Show the field ONLY if: [tipo_enfermedad2] = '3'	Especificar	radio<br>1 Aneurisma coronario<br>2 Infarto<br>3 Cardiopatía isquémica en < 45 años<br>4 Muerte súbita de causa cardiológica en < 45 años<br>5 Historia previa de aneurismas familiares<br>6 Otros (especificar)<br><br>Custom alignment: LV
53	enf_cardiaca_otra2<br>Show the field ONLY if: [enfer_cardiaca2] = '6'	Especificar	text
54	infeccion_foco2<br>Show the field ONLY if: [tipo_enfermedad2] = '6'	Foco	radio<br>1 ORL<br>2 Infección respiratoria de vías bajas<br>3 Neumonía<br>4 Infección del tracto urinario<br>5 Meningitis<br>6 Infección de piel y partes blandas<br>7 Sepsis/bacteriemia<br>8 Otros (especificar)<br><br>Custom alignment: LV
55	infeccion_foco_otro2<br>Show the field ONLY if: [infeccion_foco2] = '8'	Especificar	text
56	infeccion_patogeno2<br>Show the field ONLY if: [tipo_enfermedad2] = '6'	¿Se identificó patógeno?	radio<br>1 Si<br>0 No<br><br>Custom alignment: RH
57	infeccion_patogeno_espe2<br>Show the field ONLY if: [infeccion_patogeno2] = '1'	Especificar	text
58	comentarios_2	Comentarios	notes<br>Custom alignment: LV
59		Complete?	dropdown

	datos_sociodemogrficos_complete		0 Incomplete<br>1 Unverified<br>2 Complete
Instrument: **Antropometria**			
60	peso	Peso<br>*_ _ . _ kg*	text (number_1dp)
61	talla	Talla o longitud<br>*_ _ _ cm*	text (integer)
62	sc	SC<br>*kg/m^2*	calc<br>Calculation: round([peso]/(([talla]/100) * ([talla]/100)), 2)
63	comentarios_3	Comentarios	notes<br>Custom alignment: LV
64	antropometria_complete	Complete?	dropdown<br>0 Incomplete<br>1 Unverified<br>2 Complete
Instrument: **Datos clínicos**			
65	diagnostico_kawasaki	Diagnóstico de enfermedad de Kawasaki<br>Introduzca también los casos de diagnóstico tardío que no recibieron tratamiento<br>Se refiere al diagnóstico final del caso después de un seguimiento mínimo de 6 semanas, para confirmar si aparecen descamación característica de dedos de las manos o pies, trombocitosis o alteraciones coronarias (mínimo 2 ecografías en ese periodo).	radio<br>1 Completo (ver criterios diagnósticos en apartado preliminaries)<br>2 Incompleto (ver criterios diagnósticos en apartado preliminaries)<br>3 Atípico (ver criterios diagnósticos en apartado preliminaries)<br>4 Sospecha de Enfermedad de Kawasaki, pero no cumple ninguno de los previos<br>Custom alignment: LV
66	sospecha_otra_enfer	A pesar de que tenga criterios de Enfermedad de Kawasaki y se maneje como tal, ¿tiene la sospecha de que se pueda tratar de otra enfermedad?	radio<br>1 Si<br>0 No<br>Custom alignment: LH
67	otra_enfermedad<br>Show the field ONLY if: [sospecha_otra_enfer] = '1'	Cuál	radio<br>1 Shock tóxico<br>2 Síndrome hemofagocítico (ver criterios diagnósticos en apartado preliminaries)<br>3 Escarlatina<br>4 Infección por adenovirus<br>5 Infección por EBV<br>6 Otra<br>Custom alignment: LV
68	otra_enfermedad_espe<br>Show the field ONLY if: [otra_enfermedad] = '6'	Especificar	text
69	primer_episodio	¿Es un primer episodio?<br>Se considerará segundo episodio o Enfermedad de Kawasaki recurrente si han pasado más de dos meses del tratamiento inicial.<br>Dentro de los dos primeros meses del tratamiento inicial se considerará reagudización	radio<br>1 Si<br>0 No<br>Custom alignment: LH
70	primer_episodio_tipo<br>Show the field ONLY if: [primer_episodio] = 0		radio<br>1 EK recurrente o segundo episodio<br>2 Reagudización de EK<br>Custom alignment: LH
71	fdiagnostico_previo<br>Show the field ONLY if: [primer_episodio] = '0'	Fecha diagnostico previo<br>*(dd/mm/aaaa)*	text (date_dmy)
72	infeccion_previa	Antecedentes infecciones previas ultimas 4 últimas semanas	radio<br>1 Si<br>0 No<br>Custom alignment: RH

73	infeccion_previa_foco<br>Show the field ONLY if: [infe ccion_previa] = '1'	Foco	radio<br>1 ORL vírico<br>2 Infección respiratoria de vías bajas<br>3 Neumonía<br>4 Infección del tracto urinario<br>5 Meningitis<br>6 Infección de piel y partes blandas<br>7 Sepsis/bacteriemia<br>8 Otros (especificar)<br>Custom alignment: LV
74	infeccion_previa_foco_espe<br>Show the field ONLY if: [infe ccion_previa_foco] = '8'	Especificar	text
75	infeccion_previa_patogeno<br>Show the field ONLY if: [infe ccion_previa] = '1'	¿Se identificó patógeno?	radio<br>1 Si<br>0 No<br>Custom alignment: RH
76	infeccion_previa_patogeno_espe<br>Show the field ONLY if: [infe ccion_previa_patogeno] = '1'	Especificar	text
77	fiebre	Fiebre	radio<br>1 Si<br>0 No<br>Custom alignment: RH
78	fecha_inicio_fiebre<br>Show the field ONLY if: [fieb re] = '1'	Fecha inicio fiebre<br>Por fecha de inicio de la fiebre se entiende el primer día que notaron temperatura corporal elevada, aunque no la comprobaran con termómetro<br>*(dd/mm/aaaa)*	text (date_dmy)
79	tempartura_maxima<br>Show the field ONLY if: [fieb re] = '1'	Temperatura máxima ºC<br>Tª máxima registrada o referida<br>*_ _ . _ ºC*	text (number_1dp)
80	medida_temperatura<br>Show the field ONLY if: [fieb re] = '1'	Medida de la temperatura<br>Referido a la temperatura máxima, indique el sistema de medición utilizado.<br>Si la temperatura máxima la refirieron los padres y/o no está recogido el sistema de medición que usaron, indique "Desconocido"	radio<br>1 Axilar<br>2 Oral<br>3 Rectal<br>4 Ótico<br>99 Desconocido<br>5 Otros<br>Custom alignment: LV
81	medida_temp_otros<br>Show the field ONLY if: [me dida_temperatura] = '5'	Especificar	text
82	fiebre_duracion<br>Show the field ONLY if: [fieb re] = '1'	Duración de la fiebre<br>Se consideran días naturales, no periodos de 24 horas. Una fracción de día se considera un día.<br>Por ejemplo, si la fiebre se comprobó por primera vez el lunes a las 21 horas y la última determinación de temperatura elevada se realizó el domingo a las 8 de la mañana, la duración total de la fiebre será 7 días. Si el domingo previo "le notaron caliente" pero no comprobaron la temperatura, la duración total será 8 días<br>*días*	text (integer)<br>Custom alignment: LV
83	resolucion_fiebre<br>Show the field ONLY if: [fieb re] = '1'	¿La resolución de la fiebre coincide con tratamiento para EK?	radio<br>1 Si<br>0 No<br>Custom alignment: RH
84	sintoma_antes_fiebre<br>Show the field ONLY if: [fieb re] = '1'	¿Presentó algún síntoma sugestivo de EK antes del inicio de la fiebre?	radio<br>1 Si<br>0 No<br>Custom alignment: RH
85			radio<br>1 Inyección conjuntival

	sintoma_antes_fiebre_espe<br>Show the field ONLY if: [sintoma_antes_fiebre] = '1'		2 Alteraciones cavidad oral y/o labios<br>3 Afectación de manos y/o pies<br>4 Exantema polimorfo, (sin ampollas ni vesículas)<br>5 Adenopatías<br>6 Otros (especificar)<br><br>Custom alignment: LV
86	sintoma_antes_fiebre_otro<br>Show the field ONLY if: [sintoma_antes_fiebre_espe] = 6	Especificar	text
87	sintoma_antes_fiebre_fecha<br>Show the field ONLY if: [sintoma_antes_fiebre] = '1'	Fecha incio<br>*(dd/mm/aaaa)*	text (date_dmy)
88	inyeccion_conjuntival	Inyección conjuntival	radio<br>1 Si<br>0 No<br><br>Custom alignment: RH
89	alt_cavidad_oral	Alteraciones cavidad oral y/o labios	radio<br>1 Si<br>0 No<br><br>Custom alignment: RH
90	alt_cavidad_oral_espe<br>Show the field ONLY if: [alt_cavidad_oral] = '1'	Especificar	checkbox<br>1 alt_cavidad_oral_espe___1 Fisura labial<br>2 alt_cavidad_oral_espe___2 Lengua de fresa<br>3 alt_cavidad_oral_espe___3 Faringitis<br><br>Custom alignment: LV
91	alt_extremidades	Afectación de manos y/o pies	radio<br>1 Si<br>0 No<br><br>Custom alignment: RH
92	alt_extremidades_espe<br>Show the field ONLY if: [alt_extremidades] = '1'	Especificar	checkbox<br>1 alt_extremidades_espe___1 Edema que afecta principalmente a dedos<br>2 alt_extremidades_espe___2 Eritema difuso en palmas y/o plantas<br>3 alt_extremidades_espe___3 Descamación característica (de inicio subungueal, extendiéndose después por las yemas de los dedos y palmas/plantas, sin sobrepasar la muñeca)<br><br>Custom alignment: LV
93	fecha_inicio_descamacion<br>Show the field ONLY if: [alt_extremidades_espe(3)] = '1'	Fecha inicio Descamación<br>*(dd/mm/aaaa)*	text (date_dmy)
94	exantema_polimorfo	Exantema sin vesículas ni ampollas	radio<br>1 Si<br>0 No<br><br>Custom alignment: RH
95	adenopatias	Adenopatías<br>Un conglomerado adenopático (por palpación o por ecografía) de tamaño superior a 1,5 cm equivale a una adenopatía única de ese tamaño	radio<br>1 Si<br>0 No<br><br>Custom alignment: RH
96	adenopatia_espe<br>Show the field ONLY if: [adenopatias] = '1'	Especificar	radio<br>1 Única > 1.5cm

			2 Única < 1.5cm<br>3 Múltiples<br><br>Custom alignment: LV
97	adenopatia_localizacion<br>Show the field ONLY if: [adenopatia_espe] = '1' or [adenopatia_espe] = '2'	Localización	radio<br>1 Cervical<br>2 Intraparotidea<br>3 Axilar<br>4 Inguinal<br>5 Poplitea<br>6 Otros (especificar)<br><br>Custom alignment: LV
98	adenopatia_localizacion_espe<br>Show the field ONLY if: [adenopatia_localizacion] = '6'	Especificar	text
99	inflamacion_cicatriz	Inflamación/eritema en cicatriz BCG	radio<br>1 Si<br>0 No<br>99 Se desconoce<br><br>Custom alignment: RH
100	exantema_eritema	Exantema/eritema difuso en área del pañal	radio<br>1 Si<br>0 No<br>99 Se desconoce<br><br>Custom alignment: RH
101	irritabilidad	Irritabilidad	radio<br>1 Si<br>0 No<br>99 Se desconoce<br><br>Custom alignment: RH
102	meningitis_aseptica	Meningitis aséptica/Encefalitis antes de administrar IVIG	radio<br>1 Si<br>0 No<br>99 Se desconoce<br><br>Custom alignment: RH
103	sint_digetivos	Síntomas digestivos: Dolor abdominal, náuseas y vómitos	radio<br>1 Si<br>0 No<br>99 Se desconoce<br><br>Custom alignment: RH
104	sint_osteoarticulares	Síntomas osteoarticulares: Artralgias/Artritis antes del tratamiento	radio<br>1 Si<br>0 No<br>99 Se desconoce<br><br>Custom alignment: RH
105	alt_hepaticas	Alteraciones hepáticas	radio<br>1 Si<br>0 No<br>99 Se desconoce<br><br>Custom alignment: RH
106	alt_hepaticas_espe<br>Show the field ONLY if: [alt_hepaticas] = '1'	Especificar	checkbox<br>1 alt_hepaticas_espe___1 Elevación de transaminasas<br>2 alt_hepaticas_espe___2 Elevación de la Bilirrubina Total<br>3 alt_hepaticas_espe___3

			Elevación del Colesterol<br>4 | alt_hepaticas_espe___4 | Otros (especificar)<br><br>Custom alignment: LV
107	alt_hepaticas_otras<br>Show the field ONLY if: [alt_hepaticas_espe(4)] = '1'	Especificar	text
108	aneurismas_vasos	Aneurismas en vasos periféricos<br>Indique Si si los detectó.<br>Indique Se desconoce si no realizó una prueba de imagen para buscarlos.<br>Indique No si realizó en los dos primeros meses una prueba de imagen para buscarlos y no los halló (Ej: angioTC o angioRM)	radio<br>1 | Si<br>0 | No<br>99 | Se desconoce<br><br>Custom alignment: RH
109	aneurismas_prueba<br>Show the field ONLY if: [aneurismas_vasos] = '1' or [aneurismas_vasos] = '0'	Especificar prueba realizada	radio<br>1 | Ecografia/ecografía doppler<br>2 | AngioTC<br>3 | AngioRM<br>4 | Otra (especificar)<br><br>Custom alignment: LV
110	aneurismas_prueba_espe<br>Show the field ONLY if: [aneurismas_prueba] = '4'	Especificar	text
111	shock	¿Ha presentado en paciente hipotensión o shock?	radio<br>1 | Si<br>0 | No<br>99 | Se desconoce<br><br>Custom alignment: RH
112	shock_tas<br>Show the field ONLY if: [shock] = '1'	TAS < P5 para la edad, descenso mantenido de la TA sistólica un 20% respecto a los valores iniciales o signos de mala perfusión	radio<br>1 | Si<br>0 | No<br>99 | Se desconoce<br><br>Custom alignment: RH
113	shock_no_infusion<br>Show the field ONLY if: [shock] = 1	¿Presentó alguno de estos signos cuando NO recibía una infusión de IgG?	radio<br>1 | Si<br>0 | No<br>99 | Se desconoce<br><br>Custom alignment: RH
114	shock_durante_infusion<br>Show the field ONLY if: [shock] = 1	¿Presentó alguno de estos signos DURANTE una infusión de IgG?	radio<br>1 | Si<br>0 | No<br>99 | Se desconoce<br><br>Custom alignment: RH
115	shock_requirio<br>Show the field ONLY if: [shock] = 1	Requirió	checkbox<br>1 | shock_requirio___1 | Ningún tratamiento específico<br>2 | shock_requirio___2 | Reducir ritmo de infusión de IgG<br>3 | shock_requirio___3 | Expansión de volumen<br>4 | shock_requirio___4 | Drogas vasoactivas<br>5 | shock_requirio___5 | Ingreso en UCI<br><br>Custom alignment: LV
116	pancreatitis	Pancreatitis	radio<br>1 | Si<br>0 | No<br>99 | Se desconoce<br><br>Custom alignment: RH
117	uveitis	A parte de la hiperemia conjuntival inicial, ¿ha presentado el paciente alguna afectación ocular?	radio<br>1 | Si

			0 No<br>99 Se desconoce<br><br>Custom alignment: RH
118	uveitis_tipo<br>Show the field ONLY if: [uveitis] = '1'	Tipo	radio<br>1 Uveítis anterior transitoria<br>2 Queratitis<br>3 Afectación ocular persistente (especificar)<br>4 Otros (especificar)<br><br>Custom alignment: LV
119	uveitis_tipo_espe<br>Show the field ONLY if: [uveitis_tipo] = '3' or [uveitis_tipo] = '4'	Especificar	text
120	hidrops_vesicular	Hidrops vesicular	radio<br>1 Si<br>0 No<br>99 Se desconoce<br><br>Custom alignment: RH
121	sordera_neurosensorial	Hipoacusia o Sordera neurosensorial	radio<br>1 Si<br>0 No<br>99 Se desconoce<br><br>Custom alignment: RH
122	sordera_fecha<br>Show the field ONLY if: [sordera_neurosensorial] = '1'	Fecha de realización de prueba de detección<br>*(dd/mm/aaaa)*	text (date_dmy)
123	sordera_grado<br>Show the field ONLY if: [sordera_neurosensorial] = '1'	Grado afectación	text
124	broncoespasmo	Broncoespasmo	radio<br>1 Si<br>0 No<br>99 Se desconoce<br><br>Custom alignment: RH
125	hematuria	Hematuria	radio<br>1 Si<br>0 No<br>99 Se desconoce<br><br>Custom alignment: RH
126	otros_sintomas	Otros	radio<br>1 Si<br>0 No<br><br>Custom alignment: RH
127	otros_signos_sintomas<br>Show the field ONLY if: [otros_sintomas] = '1'	Especificar	text
128	comentarios_4	Comentarios	notes<br>Custom alignment: LV
129	datos_clnicos_complete	Complete?	dropdown<br>0 Incomplete<br>1 Unverified<br>2 Complete
Instrument: **Datos analíticos**			
130	hemoglobina	Hemoglobina<br>MÍNIMA, en cualquier hemograma, antes del tratamiento<br>*g/dl*	text (number)
131	hemoglobina_fecha	Fecha<br>*(dd/mm/aaaa)*	text (date_dmy)
132	plaquetas	Plaquetas<br>MÍNIMA, en cualquier hemograma	text (number)

		X10^9/L	
133	plaquetas_fecha	Fecha<br>*(dd/mm/aaaa)*	text (date_dmy)
134	plaquetas3	Plaquetas<br>MÁXIMA, en todo el seguimiento<br>*X10^9/L*	text (number)
135	plaquetas_fecha3	Fecha<br>*(dd/mm/aaaa)*	text (date_dmy)
136	leucocitos	Leucocitos totales<br>MÁXIMA, en cualquier hemograma, en el periodo agudo (4-6 semanas desde inicio de los síntomas)<br>*X10^9/L*	text (number)
137	leucocitos_fecha	Fecha<br>*(dd/mm/aaaa)*	text (date_dmy)
138	neutrofilos	Neutrófilos totales<br>MÁXIMA, en cualquier hemograma, en el periodo agudo (4-6 semanas desde inicio de los síntomas)<br>Puede no ser del mismo hemograma que los otros parámetros del recuento de leucocitos<br>*X10^9/L*	text (number)
139	neutrofilos_fecha	Fecha<br>*(dd/mm/aaaa)*	text (date_dmy)
140	neutrofilos_porcentaje	Neutrófilos %<br>MÁXIMA, en cualquier hemograma, en el periodo agudo (4-6 semanas desde inicio de los síntomas)<br>Puede no ser del mismo hemograma que los otros parámetros del recuento de leucocitos<br>*%*	text (number)
141	neutrofilos_porcentaje_fech a	Fecha<br>*(dd/mm/aaaa)*	text (date_dmy)
142	eosinofilos_totales	Eosinófilos totales<br>MÁXIMA en el periodo agudo (4-6 semanas desde inicio de los síntomas)<br>*X10^9/L*	text (number)
143	eosinofilos_totales_fecha	Fecha<br>*(dd/mm/aaaa)*	text (date_dmy)
144	vsg	VSG<br>MÁXIMA en el periodo agudo (4-6 semanas desde inicio de los síntomas)<br>*mm/h*	text (number)
145	vsg_fecha	Fecha<br>*(dd/mm/aaaa)*	text (date_dmy)
146	sodio	Sodio<br>MÍNIMO en el periodo agudo (4-6 semanas desde inicio de los síntomas)<br>Revise todas las analíticas previas a IVIG, aunque sean de bastantes días antes o de otro centro sanitario<br>*meq/l*	text (number)
147	sodio_fecha	Fecha<br>*(dd/mm/aaaa)*	text (date_dmy)
148	creatinina	Creatinina<br>MÁXIMA en el periodo agudo (4-6 semanas desde inicio de los síntomas)<br>*mg/dm*	text (number)
149	creatinina_fecha	Fecha<br>*(dd/mm/aaaa)*	text (date_dmy)
150	bilirrubina	Bilirrubina Total<br>MÁXIMA en el periodo agudo (4-6 semanas desde inicio de los síntomas)<br>Revise todas las analíticas, especialmente las de los primeros días de fiebre<br>*mg/dl*	text (number)
151	bilirrubina_fecha	Fecha<br>*(dd/mm/aaaa)*	text (date_dmy)
152	bilirrubina2	Bilirrubina Directa<br>MÁXIMA en el periodo agudo (4-6 semanas desde inicio de los síntomas)<br>Revise todas las analíticas, especialmente las de los primeros días de fiebre<br>*mg/dl*	text (number)
153	bilirrubina2_fecha	Fecha<br>*(dd/mm/aaaa)*	text (date_dmy)
154	albumina2	Albumina<br>MÍNIMA en el periodo agudo (4-6 semanas desde inicio de los síntomas)<br>*g/L*	text (number)
155	albumina2_fecha	Fecha<br>*(dd/mm/aaaa)*	text (date_dmy)
156	ast		text (number)

		GOT/ASAT<br>MÁXIMA en el periodo agudo (4-6 semanas desde inicio de los síntomas)<br>*IU/dl*	
157	ast_fecha	Fecha<br>*(dd/mm/aaaa)*	text (date_dmy)
158	alt	GPT/ALAT<br>MÁXIMA en el periodo agudo (4-6 semanas desde inicio de los síntomas)<br>*IU/L*	text (number)
159	alt_fecha	Fecha<br>*(dd/mm/aaaa)*	text (date_dmy)
160	pcr	PCR<br>MÁXIMA en el periodo agudo (4-6 semanas desde inicio de los síntomas)<br>Revise todas las analíticas, especialmente las de los primeros días de fiebre<br>*mg/dl*	text (number)
161	pcr_fecha	Fecha<br>*(dd/mm/aaaa)*	text (date_dmy)
162	pct	Procalcitonina (PCT)	radio<br>1 Realizado<br>0 No Realizado<br>Custom alignment: RH
163	pct2<br>Show the field ONLY if: [pct] = '1'	Procalcitonina (PCT)<br>MÁXIMA en el periodo agudo (4-6 semanas desde inicio de los síntomas)<br>*ng/ml*	text (number)
164	pct2_fecha<br>Show the field ONLY if: [pct] = 1	Fecha<br>*(dd/mm/aaaa)*	text (date_dmy)
165	ferritina	Ferritina	radio<br>1 Realizado<br>0 No Realizado<br>Custom alignment: RH
166	ferritina2<br>Show the field ONLY if: [ferritina] = '1'	Ferritina<br>MÁXIMA en el periodo agudo (4-6 semanas desde inicio de los síntomas)<br>*ng/ml*	text (number)
167	ferritina2_fecha<br>Show the field ONLY if: [ferritina] = '1'	Fecha<br>*(dd/mm/aaaa)*	text (date_dmy)
168	fibrinogeno	Fibrinógeno	radio<br>1 Realizado<br>0 No Realizado<br>Custom alignment: RH
169	fibrinogeno2<br>Show the field ONLY if: [fibrinogeno] = 1	Fibrinógeno<br>MÍNIMO en el periodo agudo (4-6 semanas desde inicio de los síntomas)<br>*mg/dl*	text (number)
170	fibrinogeno2_fecha<br>Show the field ONLY if: [fibrinogeno] = 1	Fecha<br>*(dd/mm/aaaa)*	text (date_dmy)
171	cpk	CPK	radio<br>1 Realizado<br>0 No Realizado<br>Custom alignment: RH
172	cpk2<br>Show the field ONLY if: [cpk] = '1'	CPK<br>MÁXIMA en el periodo agudo (4-6 semanas desde inicio de los síntomas)<br>*UI/mm^3*	text (number)
173	cpk2_fecha<br>Show the field ONLY if: [cpk] = '1'	Fecha<br>*(dd/mm/aaaa)*	text (date_dmy)
174	cpk_mb	CPK-MB	radio<br>1 Realizado<br>0 No Realizado<br>Custom alignment: RH
175			text (number)

	cpk_mb2<br>Show the field ONLY if: [cpk_mb] = 1	CPK-MB<br>MÁXIMA en el periodo agudo (4-6 semanas desde inicio de los síntomas)<br>*UI/mm^3*	
176	cpk_mb2_fecha<br>Show the field ONLY if: [cpk_mb] = 1	Fecha<br>*(dd/mm/aaaa)*	text (date_dmy)
177	troponina_t	Troponina T	radio<br>1 Realizado<br>0 No Realizado<br><br>Custom alignment: RH
178	troponina_t2<br>Show the field ONLY if: [troponina_t] = 1	Troponina T<br>MÁXIMA en el periodo agudo (4-6 semanas desde inicio de los síntomas<br>*UI/mm^3*	text (number)
179	troponina_t2_fecha<br>Show the field ONLY if: [troponina_t] = 1	Fecha<br>*(dd/mm/aaaa)*	text (date_dmy)
180	ldh	LDH	radio<br>1 Realizado<br>0 No Realizado<br><br>Custom alignment: RH
181	ldh2<br>Show the field ONLY if: [ldh] = 1	LDH<br>MÁXIMA en el periodo agudo (4-6 semanas desde inicio de los síntomas)<br>*UI/L*	text (number)
182	ldh2_fecha<br>Show the field ONLY if: [ldh] = 1	Fecha<br>*(dd/mm/aaaa)*	text (date_dmy)
183	probnp	ProBNP	radio<br>1 Realizado<br>0 No Realizado<br><br>Custom alignment: RH
184	probnp2<br>Show the field ONLY if: [probnp] = '1'	ProBNP<br>MÁXIMA antes del tratamiento<br>*pg/ml*	text (number)
185	probnp2_fecha<br>Show the field ONLY if: [probnp] = '1'	Fecha<br>*(dd/mm/aaaa)*	text (date_dmy)
186	probnp3<br>Show the field ONLY if: [probnp] = '1'	ProBNP<br>MÁXIMA en el periodo agudo (4-6 semanas desde inicio de los síntomas)<br>*pg/ml*	text (number)
187	probnp3_fecha<br>Show the field ONLY if: [probnp] = '1'	Fecha<br>*(dd/mm/aaaa)*	text (date_dmy)
188	nt_probnp	NT-proBNP	radio<br>1 Realizado<br>0 No Realizado<br><br>Custom alignment: RH
189	nt_probnp2<br>Show the field ONLY if: [nt_probnp] = '1'	NT-proBNP<br>MÁXIMA antes del tratamiento<br>*pg/ml*	text (number)
190	nt_probnp2_fecha<br>Show the field ONLY if: [nt_probnp] = '1'	Fecha<br>*(dd/mm/aaaa)*	text (date_dmy)
191	nt_probnp3<br>Show the field ONLY if: [nt_probnp] = '1'	NT-proBNP<br>MÁXIMA en el periodo agudo (4-6 semanas desde inicio de los síntomas)<br>*pg/ml*	text (number)
192	nt_probnp3_fecha<br>Show the field ONLY if: [nt_probnp] = '1'	Fecha<br>*(dd/mm/aaaa)*	text (date_dmy)
193	bnp	BNP	radio<br>1 Realizado<br>0 No Realizado<br><br>Custom alignment: RH
194		BNP<br>MÁXIMA antes del tratamiento	text (number)

	bnp2<br>Show the field ONLY if: [bnp] = '1'	*pg/ml*	
195	bnp2_fecha<br>Show the field ONLY if: [bnp] = '1'	Fecha<br>*(dd/mm/aaaa)*	text (date_dmy)
196	bnp3<br>Show the field ONLY if: [bnp] = '1'	BNP<br>MÁXIMA en el periodo agudo (4-6 semanas desde inicio de los síntomas)<br>*pg/ml*	text (number)
197	bnp3_fecha<br>Show the field ONLY if: [bnp] = '1'	Fecha<br>*(dd/mm/aaaa)*	text (date_dmy)
198	piuria_orina	Piuria en sedimento de orina (antes del tratamiento)	radio<br>1 Si<br>0 No<br>99 Desconocido<br><br>Custom alignment: RH
199	piuria_orina_metodo<br>Show the field ONLY if: [piuria_orina] = 1		radio<br>1 Detectado en sedimento<br>2 Detectado en tira de orina<br>3 Detectado en sedimento y tira de orina<br><br>Custom alignment: LV
200	comentarios_5	Comentarios	notes<br>Custom alignment: LV
201	datos_analticos_complete	Complete?	dropdown<br>0 Incomplete<br>1 Unverified<br>2 Complete
Instrument: **Exploración cardiaca**			
202	ecocardiograma		radio<br>1 Si<br>0 No<br><br>Custom alignment: RH
203	ecocardiograma_tto<br>Show the field ONLY if: [ecocardiograma] = '1'		radio<br>1 Antes de inicio de tratamiento medico<br>2 Después de inicio de tratamiento medico<br><br>Custom alignment: LH
204	eco_alteraciones<br>Show the field ONLY if: [ecocardiograma] = '1'	Alteraciones	radio<br>1 Si<br>0 No<br>99 Desconocido<br><br>Custom alignment: RH
205	afectacion_coronaria<br>Show the field ONLY if: [eco_alteraciones] = '1'	Afectación coronaria	radio<br>1 Si<br>0 No<br>99 Desconocido<br><br>Custom alignment: RH
206	grados_dilatacion<br>Show the field ONLY if: [afectacion_coronaria] = '1'	Grados de dilatación coronaria<br><br>Se utilizarán como valores normales los publicados por Delaire y Dahdah (conocidos como Z-Score de Montreal) y se utilizarán los siguientes criterios:<br><br> No dilatación coronaria: Z-score < 2<br><br> Ectasia o dilatación: el máximo Z-score ≥ 2 y < 2.5<br><br> Aneurisma pequeño a moderado: Z-score ≥ 2.5 y el diámetro interno < 5 mm<br><br> Aneurisma	descriptive

		grande: Z-score ≥ 5 y < 10 y el diámetro interno < 8 mm<br><br> Aneurisma gigante: Z-score ≥ 10 y o el diámetro interno ≥ 8 mm<br><br>Disponible una calculadora en: http://parameterz.blogspot.com.es/2010/11/montreal-coronary-artery-z-scores.html	
207	hiperrefringencia<br>Show the field ONLY if: [afectacion_coronaria] = '1'	Hiperrefringencia	radio<br>1 Si<br>0 No<br>99 Desconocido<br><br>Custom alignment: RH
208	hiperrrefringencia_evol<br>Show the field ONLY if: [hiperrefringencia] = '1'	Evolución	radio<br>1 Transitoria<br>2 Persistente (mas de 6-8 semanas de enfermedad)<br>3 Desconocido<br><br>Custom alignment: LV
209	ectasia<br>Show the field ONLY if: [afectacion_coronaria] = '1'	Ectasia	radio<br>1 Si<br>0 No<br>99 Desconocido<br><br>Custom alignment: RH
210	ectasia_localizacion<br>Show the field ONLY if: [ectasia] = '1'	Localización	checkbox<br>1 ectasia_localizacion___1 Arteria coronaria principal izquierda = LCA<br>2 ectasia_localizacion___2 Circunfleja = CX<br>3 ectasia_localizacion___3 Descendente anterior izquierda = LAD<br>4 ectasia_localizacion___4 Arteria coronaria derecha = RCA<br>5 ectasia_localizacion___5 Otras (especificar)<br><br>Custom alignment: LV
211	ectasia_localizacion_otra<br>Show the field ONLY if: [ectasia_localizacion(5)] = '1'	Especificar	text
212	ectasia_evol<br>Show the field ONLY if: [ectasia] = '1'	Evolución	radio<br>1 Transitoria<br>2 Persistente (mas de 6-8 semanas de enfermedad)<br>3 Desconocido<br><br>Custom alignment: LV
213	aneurismas<br>Show the field ONLY if: [afectacion_coronaria] = '1'	Aneurismas	radio<br>1 Si<br>0 No<br>99 Desconocido<br><br>Custom alignment: RH
214	aneurismas_numero<br>Show the field ONLY if: [aneurismas] = '1'	Numero de aneurismas	text (integer)
215	aneurismas_tamano<br>Show the field ONLY if: [aneurismas] = '1'	Tamaño de aneurisma mayor<br>*mm*	text (number)
216	aneurismas_zscore<br>Show the field ONLY if: [aneurismas] = '1'	Z score aneurisma mayor (Clasificación Montreal)	radio<br>1 Aneurisma pequeño a moderado: Z-score ≥2.5 y el diámetro interno < 5 mm<br>2 Aneurisma grande: Z-score ≥5 y < 10 y el diámetro interno < 8 mm<br>3 Aneurisma gigante: Z-score ≥10 y el diámetro interno ≥8 mm<br><br>Custom alignment: LV

217	aneurismas_vasos_afectados<br>Show the field ONLY if: [aneurismas] = '1'	Número de vasos afectados	text (number)
218	aneurismas_localizacion<br>Show the field ONLY if: [aneurismas] = '1'	Localización	checkbox<br>1 aneurismas_localizacion___1 Arteria coronaria principal izquierda = LCA<br>2 aneurismas_localizacion___2 Circunfleja = CX<br>3 aneurismas_localizacion___3 Descendente anterior izquierda = LAD<br>4 aneurismas_localizacion___4 Arteria coronaria derecha = RCA<br>5 aneurismas_localizacion___5 Otras (especificar)<br>Custom alignment: LV
219	aneurismas_localizacion_otra<br>Show the field ONLY if: [aneurismas_localizacion(5)] = '1'	Especificar	text
220	aneurismas_evol<br>Show the field ONLY if: [aneurismas] = '1'	Evolución	radio<br>1 Transitoria<br>2 Persistente (mas de 6-8 semanas de enfermedad)<br>3 Desconocido<br>Custom alignment: LV
221	aneurismas_resul<br>Show the field ONLY if: [aneurismas] = '1'	Resolución completa	radio<br>1 Si<br>0 No<br>99 Desconocido<br>Custom alignment: RH
222	aneurismas_semanas<br>Show the field ONLY if: [aneurismas_resul] = '1'	Semanas desde inicio de enfermedad<br>*semanas*	text (integer)
223	estenosis<br>Show the field ONLY if: [afectacion_coronaria] = '1'	Estenosis	radio<br>1 Si<br>0 No<br>99 Desconocido<br>Custom alignment: RH
224	estenosis_localizacion<br>Show the field ONLY if: [estenosis] = '1'	Localización	checkbox<br>1 estenosis_localizacion___1 Arteria coronaria principal izquierda = LCA<br>2 estenosis_localizacion___2 Circunfleja = CX<br>3 estenosis_localizacion___3 Descendente anterior izquierda = LAD<br>4 estenosis_localizacion___4 Arteria coronaria derecha = RCA<br>5 estenosis_localizacion___5 Otras (especificar)<br>Custom alignment: LV
225	estenosis_localizacion_otra<br>Show the field ONLY if: [estenosis_localizacion(5)] = '1'	Especificar	text
226	angiotac<br>Show the field ONLY if: [afectacion_coronaria] = '1'	AngioTAC	radio<br>1 Si<br>0 No<br>99 Desconocido

			Custom alignment: RH
227	angiotac_semanas<br>Show the field ONLY if: [angiotac] = 1	Semanas desde inicio de enfermedad<br>*semanas*	text (integer)
228	angiotac_resul<br>Show the field ONLY if: [angiotac] = 1	Resultado	notes<br>Custom alignment: LV
229	coronariografia<br>Show the field ONLY if: [afectacion_coronaria] = '1'	Coronariografía	radio<br>1 Si<br>0 No<br>99 Desconocido<br>Custom alignment: RH
230	coronariografia_semanas<br>Show the field ONLY if: [coronariografia] = 1	Semanas desde inicio de enfermedad<br>*semanas*	text (integer)
231	coronariografia_resul<br>Show the field ONLY if: [coronariografia] = 1	Resultado	notes<br>Custom alignment: LV
232	derrame_pericardico<br>Show the field ONLY if: [eco_alteraciones] = '1'	Derrame pericárdico	radio<br>1 Si<br>0 No<br>99 Desconocido<br>Custom alignment: RH
233	derrame_pericardico_gravedad<br>Show the field ONLY if: [derrame_pericardico] = 1	Gravedad	radio<br>1 Leve (incluye mínimo derrame e hiperrefringencia pericárdica)<br>2 Moderado<br>3 Grave<br>Custom alignment: LV
234	derrame_pericardico_max<br>Show the field ONLY if: [derrame_pericardico] = '1'	Máximo derrame<br>*mm*	text (number)
235	derrame_pericardico_evol<br>Show the field ONLY if: [derrame_pericardico] = 1	Evolución	radio<br>1 Transitoria<br>2 Persistente (mas de 6-8 semanas de enfermedad)<br>3 Desconocido<br>Custom alignment: LV
236	alteracion_valvular<br>Show the field ONLY if: [eco_alteraciones] = '1'	Alteración valvular	radio<br>1 Si<br>0 No<br>99 Desconocido<br>Custom alignment: RH
237	alteracion_valvular_tipo<br>Show the field ONLY if: [alteracion_valvular] = 1		checkbox<br>1 alteracion_valvular_tipo___1 Insuficiencia mitral<br>2 alteracion_valvular_tipo___2 Insuficiencia tricuspide<br>3 alteracion_valvular_tipo___3 Insuficiencia aortica<br>4 alteracion_valvular_tipo___4 Insuficiencia pulmonar<br>Custom alignment: LV
238	insuficiencia_mitral_grado<br>Show the field ONLY if: [alteracion_valvular_tipo(1)] = '1'	Insuficiencia mitral	radio (Matrix)<br>1 Leve<br>2 Moderada<br>3 Severa
239	insuficiencia_tricuspide_grado<br>Show the field ONLY if: [alteracion_valvular_tipo(2)] = '1'	Insuficiencia tricuspide	radio (Matrix)<br>1 Leve<br>2 Moderada<br>3 Severa

240	insuficiencia_aortica_grado<br>Show the field ONLY if: [alte racion_valvular_tipo(3)] = '1'	Insuficiencia aortica	radio (Matrix)<br>1 Leve<br>2 Moderada<br>3 Severa
241	insuficiencia_pulmonar_grad o<br>Show the field ONLY if: [alte racion_valvular_tipo(4)] = '1'	Insuficiencia pulmonar	radio (Matrix)<br>1 Leve<br>2 Moderada<br>3 Severa
242	insuficiencia_mitral_evol<br>Show the field ONLY if: [alte racion_valvular_tipo(1)] = '1'	Insuficiencia mitral	radio (Matrix)<br>1 Transitoria<br>2 Persistente (mas de 6-8 semanas de enfermedad)<br>3 Desconocido
243	insuficiencia_tricuspide_evol<br>Show the field ONLY if: [alte racion_valvular_tipo(2)] = '1'	Insuficiencia tricuspide	radio (Matrix)<br>1 Transitoria<br>2 Persistente (mas de 6-8 semanas de enfermedad)<br>3 Desconocido
244	insuficiencia_aortica_evol<br>Show the field ONLY if: [alte racion_valvular_tipo(3)] = '1'	Insuficiencia aortica	radio (Matrix)<br>1 Transitoria<br>2 Persistente (mas de 6-8 semanas de enfermedad)<br>3 Desconocido
245	insuficiencia_pulmonar_evol<br>Show the field ONLY if: [alte racion_valvular_tipo(4)] = '1'	Insuficiencia pulmonar	radio (Matrix)<br>1 Transitoria<br>2 Persistente (mas de 6-8 semanas de enfermedad)<br>3 Desconocido
246	disminucion_contractilidad<br>Show the field ONLY if: [eco _alteraciones] = '1'	Disminución de la contractilidad	radio<br>1 Si<br>0 No<br>99 Desconocido<br>Custom alignment: RH
247	disfuncion_sistolica<br>Show the field ONLY if: [dis minucion_contractilidad] = '1'	Disfunción sistólica de VI	radio<br>1 Si<br>0 No<br>Custom alignment: RH
248	disfuncion_sistolica_fraccion<br>Show the field ONLY if: [disf uncion_sistolica] = '1'	Fracción acortamiento del VI<br>%	text (number)
249	disfuncion_sistolica_evol<br>Show the field ONLY if: [disf uncion_sistolica] = 1	Evolución	radio<br>1 Transitoria<br>2 Persistente (mas de 6-8 semanas de enfermedad)<br>3 Desconocido<br>Custom alignment: LV
250	disfuncion_segmentaria<br>Show the field ONLY if: [dis minucion_contractilidad] = '1'	Disfunción segmentaria	radio<br>1 Si<br>0 No<br>Custom alignment: RH
251	disfuncion_segmentaria_loc alizacion<br>Show the field ONLY if: [disf uncion_segmentaria] = '1'	Localización	radio<br>1 Anteroapical (septo, apex y regiones anteroseptales)<br>2 Inferobasal<br>3 Pared lateral<br>4 Ventriculo derecho<br>Custom alignment: LV
252		Evolución	radio

	disfuncion_segmentaria_evol<br>Show the field ONLY if: [disfuncion_segmentaria] = 1		1 Transitoria<br>2 Persistente (mas de 6-8 semanas de enfermedad)<br>3 Desconocido<br>Custom alignment: LV
253	ecg_realizado	Realizado	radio<br>1 Si<br>0 No<br>99 Desconocido<br>Custom alignment: RH
254	ecg_tto<br>Show the field ONLY if: [ecg_realizado] = '1'		radio<br>1 Antes de inicio de tratamiento medico<br>2 Después de inicio de tratamiento medico<br>Custom alignment: LH
255	ecg_escaneado<br>Show the field ONLY if: [ecg_realizado] = 1	Adjuntar escaneado ECG periodo agudo	file<br>Custom alignment: LV
256	ecg_escaneado2	Adjuntar escaneado al menos uno en periodo de recuperación (6-8 semanas) o posterior e indicar la semana de obtención desde el inicio de enfermedad	file<br>Custom alignment: LV
257	ecg_escaneado_semanas<br>Show the field ONLY if: [ecg_realizado] = '1'	Semanas de inicio de enfermedad<br>*semanas*	text (integer)
258	ecg_alteraciones<br>Show the field ONLY if: [ecg_realizado] = 1	Alteraciones	radio<br>1 Bajo voltaje<br>2 Alteraciones del ritmo<br>3 Bloqueos de rama<br>4 Bloqueos de la conducción AV<br>5 Alteración de la repolarización<br>Custom alignment: LV
259	ecg_descripcion<br>Show the field ONLY if: [ecg_realizado] = 1	Descripción	notes<br>Custom alignment: LV
260	ecg_evol<br>Show the field ONLY if: [ecg_realizado] = 1	Evolución	radio<br>1 Transitoria<br>2 Persistente (mas de 6-8 semanas de enfermedad)<br>3 Desconocido<br>Custom alignment: LV
261	comentarios_6	Comentarios	notes<br>Custom alignment: LV
262	exploracin_cardiaca_d48b_complete	Complete?	dropdown<br>0 Incomplete<br>1 Unverified<br>2 Complete
Instrument: **Exploración cardiaca**			
263	ecocardiograma2		radio<br>1 Si<br>0 No<br>99 Desconocido<br>Custom alignment: RH
264	exploracion_alteraciones<br>Show the field ONLY if: [ecocardiograma2] = '1'	Alteraciones	radio<br>1 Si<br>0 No<br>99 Desconocido<br>Custom alignment: RH
265	exploracion_persistencia<br>Show the field ONLY if: [exploracion_alteraciones] = '1'	Persistencia de las lesiones anteriores	radio<br>1 Si

			0 No<br>99 Desconocido<br><br>Custom alignment: RH
266	afectacion2_coronaria<br>Show the field ONLY if: [exploracion_persistencia] = '1'	Afectación coronaria	radio<br>1 Si<br>0 No<br>99 Desconocido<br><br>Custom alignment: RH
267	grados2_dilatacion<br>Show the field ONLY if: [afectacion2_coronaria] = '1'	Grados de dilatación coronaria<br><br>Se utilizarán como valores normales los publicados por Delaire y Dahdah (conocidos como Z-Score de Montreal) y se utilizarán los siguientes criterios:<br><br> No dilatación coronaria: Z-score < 2<br><br> Ectasia o dilatación: el máximo Z-score ≥ 2 y < 2.5<br><br> Aneurisma pequeño a moderado: Z-score ≥ 2.5 y el diámetro interno < 5 mm<br><br> Aneurisma grande: Z-score ≥ 5 y < 10 y el diámetro interno < 8 mm<br><br> Aneurisma gigante: Z-score ≥ 10 y o el diámetro interno ≥ 8 mm<br><br>Disponible una calculadora en: http://parameterz.blogspot.com.es/2010/11/montreal-coronary-artery-z-scores.html	descriptive
268	hiperrefringencia2<br>Show the field ONLY if: [afectacion2_coronaria] = '1'	Hiperrefringencia	radio<br>1 Si<br>0 No<br>99 Desconocido<br><br>Custom alignment: RH
269	hiperrrefringencia2_evol<br>Show the field ONLY if: [hiperrefringencia2] = '1'	Evolución	radio<br>1 Transitoria<br>2 Persistente (mas de 6-8 semanas de enfermedad)<br>3 Desconocido<br><br>Custom alignment: LV
270	ectasia2<br>Show the field ONLY if: [afectacion2_coronaria] = '1'	Ectasia	radio<br>1 Si<br>0 No<br>99 Desconocido<br><br>Custom alignment: RH
271	ectasia2_localizacion<br>Show the field ONLY if: [ectasia2] = '1'	Localización	checkbox<br>1 ectasia2_localizacion___1 Arteria coronaria principal izquierda = LCA<br>2 ectasia2_localizacion___2 Circunfleja = CX<br>3 ectasia2_localizacion___3 Descendente anterior izquierda = LAD<br>4 ectasia2_localizacion___4 Arteria coronaria derecha = RCA<br>5 ectasia2_localizacion___5 Otras (especificar)<br><br>Custom alignment: LV
272	ectasia2_localizacion_otra<br>Show the field ONLY if: [ectasia2_localizacion(5)] = '1'	Especificar	text
273	ectasia2_evol<br>Show the field ONLY if: [ectasia2] = '1'	Evolución	radio<br>1 Transitoria<br>2

			Persistente (mas de 6-8 semanas de enfermedad)<br>3 Desconocido<br><br>Custom alignment: LV
274	aneurismas2<br>Show the field ONLY if: [afectacion2_coronaria] = '1'	Aneurismas	radio<br>1 Si<br>0 No<br>99 Desconocido<br><br>Custom alignment: RH
275	aneurismas2_numero<br>Show the field ONLY if: [aneurismas2] = '1'	Numero de aneurismas	text (integer)
276	aneurismas2_tamano<br>Show the field ONLY if: [aneurismas2] = '1'	Tamaño de aneurisma mayor<br>*mm*	text (number)
277	aneurismas2_zscore<br>Show the field ONLY if: [aneurismas2] = '1'	Z score aneurisma mayor (Clasificación Montreal)	radio<br>1 Aneurisma pequeño a moderado: Z-score ≥2.5 y el diámetro interno < 5 mm<br>2 Aneurisma grande: Z-score ≥5 y < 10 y el diámetro interno < 8 mm<br>3 Aneurisma gigante: Z-score ≥10 y el diámetro interno ≥8 mm<br><br>Custom alignment: LV
278	aneurismas2_vasos_afectados<br>Show the field ONLY if: [aneurismas2] = '1'	Número de vasos afectados	text (number)
279	aneurismas2_localizacion<br>Show the field ONLY if: [aneurismas2] = '1'	Localización	checkbox<br>1 aneurismas2_localizacion___1 Arteria coronaria principal izquierda = LCA<br>2 aneurismas2_localizacion___2 Circunfleja = CX<br>3 aneurismas2_localizacion___3 Descendente anterior izquierda = LAD<br>4 aneurismas2_localizacion___4 Arteria coronaria derecha = RCA<br>5 aneurismas2_localizacion___5 Otras (especificar)<br><br>Custom alignment: LV
280	aneurismas2_localizacion_otra<br>Show the field ONLY if: [aneurismas2_localizacion(5)] = '1'	Especificar	text
281	aneurismas2_evol<br>Show the field ONLY if: [aneurismas2] = '1'	Evolución	radio<br>1 Transitoria<br>2 Persistente (mas de 6-8 semanas de enfermedad)<br>3 Desconocido<br><br>Custom alignment: LV
282	aneurismas2_resul<br>Show the field ONLY if: [aneurismas2] = '1'	Resolución completa	radio<br>1 Si<br>0 No<br>99 Desconocido<br><br>Custom alignment: RH
283	aneurismas2_semanas<br>Show the field ONLY if: [aneurismas2_resul] = '1'	Semanas desde inicio de enfermedad<br>*semanas*	text (integer)
284		Estenosis	radio<br>1 Si

	estenosis2<br>Show the field ONLY if: [afectacion2_coronaria] = '1'		0 No<br>99 Desconocido<br><br>Custom alignment: RH
285	estenosis2_localizacion<br>Show the field ONLY if: [estenosis2] = '1'	Localización	checkbox<br>1 estenosis2_localizacion___1 Arteria coronaria principal izquierda = LCA<br>2 estenosis2_localizacion___2 Circunfleja = CX<br>3 estenosis2_localizacion___3 Descendente anterior izquierda = LAD<br>4 estenosis2_localizacion___4 Arteria coronaria derecha = RCA<br>5 estenosis2_localizacion___5 Otras (especificar)<br><br>Custom alignment: LV
286	estenosis2_localizacion_otra<br>Show the field ONLY if: [estenosis2_localizacion(5)] = '1'	Especificar	text
287	angiotac2<br>Show the field ONLY if: [afectacion2_coronaria] = '1'	AngioTAC	radio<br>1 Si<br>0 No<br>99 Desconocido<br><br>Custom alignment: RH
288	angiotac2_semanas<br>Show the field ONLY if: [angiotac2] = 1	Semanas desde inicio de enfermedad<br>*semanas*	text (integer)
289	angiotac2_resul<br>Show the field ONLY if: [angiotac2] = 1	Resultado	notes<br>Custom alignment: LV
290	coronariografia2<br>Show the field ONLY if: [afectacion2_coronaria] = '1'	Coronariografía	radio<br>1 Si<br>0 No<br>99 Desconocido<br><br>Custom alignment: RH
291	coronariografia2_semanas<br>Show the field ONLY if: [coronariografia2] = 1	Semanas desde inicio de enfermedad<br>*semanas*	text (integer)
292	coronariografia2_resul<br>Show the field ONLY if: [coronariografia2] = 1	Resultado	notes<br>Custom alignment: LV
293	derrame2_pericardico<br>Show the field ONLY if: [exploracion_persistencia] = '1'	Derrame pericárdico	radio<br>1 Si<br>0 No<br>99 Desconocido<br><br>Custom alignment: RH
294	derrame2_pericardico_gravedad<br>Show the field ONLY if: [derrame2_pericardico] = 1	Gravedad	radio<br>1 Leve (incluye mínimo derrame e hiperrefringencia pericárdica)<br>2 Moderado<br>3 Grave<br><br>Custom alignment: LV
295	derrame2_pericardico_max<br>Show the field ONLY if: [derrame2_pericardico] = '1'	Máximo derrame<br>*mm*	text (number)
296	derrame2_pericardico_evol<br>Show the field ONLY if: [derrame2_pericardico] = 1	Evolución	radio<br>1 Transitoria<br>2 Persistente (mas de 6-8 semanas de enfermedad)

			3 Desconocido<br><br>Custom alignment: LV
297	alteracion2_valvular<br>Show the field ONLY if: [exploracion_persistencia] = '1'	Alteración valvular	radio<br>1 Si<br>0 No<br>99 Desconocido<br><br>Custom alignment: RH
298	alteracion2_valvular_tipo<br>Show the field ONLY if: [alteracion2_valvular] = 1		checkbox<br>1 alteracion2_valvular_tipo___1 Insuficiencia mitral<br>2 alteracion2_valvular_tipo___2 Insuficiencia tricuspide<br>3 alteracion2_valvular_tipo___3 Insuficiencia aortica<br>4 alteracion2_valvular_tipo___4 Insuficiencia pulmonar<br><br>Custom alignment: LV
299	insuficiencia2_mitral_grado<br>Show the field ONLY if: [alteracion2_valvular_tipo(1)] = '1'	Insuficiencia mitral	radio (Matrix)<br>1 Leve<br>2 Moderada<br>3 Severa
300	insuficiencia2_tricuspide_grado<br>Show the field ONLY if: [alteracion2_valvular_tipo(2)] = '1'	Insuficiencia tricuspide	radio (Matrix)<br>1 Leve<br>2 Moderada<br>3 Severa
301	insuficiencia2_aortica_grado<br>Show the field ONLY if: [alteracion2_valvular_tipo(3)] = '1'	Insuficiencia aortica	radio (Matrix)<br>1 Leve<br>2 Moderada<br>3 Severa
302	insuficiencia2_pulmonar_grado<br>Show the field ONLY if: [alteracion2_valvular_tipo(4)] = '1'	Insuficiencia pulmonar	radio (Matrix)<br>1 Leve<br>2 Moderada<br>3 Severa
303	insuficiencia2_mitral_evol<br>Show the field ONLY if: [alteracion2_valvular_tipo(1)] = '1'	Insuficiencia mitral	radio (Matrix)<br>1 Transitoria<br>2 Persistente (mas de 6-8 semanas de enfermedad)<br>3 Desconocido
304	insuficiencia2_tricuspide_evol<br>Show the field ONLY if: [alteracion2_valvular_tipo(2)] = '1'	Insuficiencia tricuspide	radio (Matrix)<br>1 Transitoria<br>2 Persistente (mas de 6-8 semanas de enfermedad)<br>3 Desconocido
305	insuficiencia2_aortica_evol<br>Show the field ONLY if: [alteracion2_valvular_tipo(3)] = '1'	Insuficiencia aortica	radio (Matrix)<br>1 Transitoria<br>2 Persistente (mas de 6-8 semanas de enfermedad)<br>3 Desconocido
306	insuficiencia2_pulmonar_evol<br>Show the field ONLY if: [alteracion2_valvular_tipo(4)] = '1'	Insuficiencia pulmonar	radio (Matrix)<br>1 Transitoria<br>2 Persistente (mas de 6-8 semanas de enfermedad)<br>3 Desconocido
307	disminucion2_contractilidad<br>Show the field ONLY if: [exploracion_persistencia] = '1'	Disminución de la contractilidad	radio<br>1 Si<br>0 No<br>99 Desconocido<br><br>Custom alignment: RH

308	disfuncion2_sistolica<br>Show the field ONLY if: [disminucion2_contractilidad] = '1'	Disfunción sistólica de VI	radio<br>1 Si<br>0 No<br><br>Custom alignment: RH
309	disfuncion2_sistolica_fraccion<br>Show the field ONLY if: [disfuncion2_sistolica] = '1'	Fracción acortamiento del VI<br>%	text (number)
310	disfuncion2_sistolica_evol<br>Show the field ONLY if: [disfuncion2_sistolica] = 1	Evolución	radio<br>1 Transitoria<br>2 Persistente (mas de 6-8 semanas de enfermedad)<br>3 Desconocido<br><br>Custom alignment: LV
311	disfuncion2_segmentaria<br>Show the field ONLY if: [disminucion2_contractilidad] = '1'	Disfunción segmentaria	radio<br>1 Si<br>0 No<br><br>Custom alignment: RH
312	disfuncion2_segmentaria_localizacion<br>Show the field ONLY if: [disfuncion2_segmentaria] = '1'	Localización	radio<br>1 Anteroapical (septo, apex y regiones anteroseptales)<br>2 Inferobasal<br>3 Pared lateral<br>4 Ventriculo derecho<br><br>Custom alignment: LV
313	disfuncion2_segmentaria_evol<br>Show the field ONLY if: [disfuncion2_segmentaria] = 1	Evolución	radio<br>1 Transitoria<br>2 Persistente (mas de 6-8 semanas de enfermedad)<br>3 Desconocido<br><br>Custom alignment: LV
314	exploracion_nuevas<br>Show the field ONLY if: [exploracion_alteraciones] = '1'	Nuevas alteraciones	radio<br>1 Si<br>0 No<br>99 Desconocido<br><br>Custom alignment: RH
315	afectacion3_coronaria<br>Show the field ONLY if: [exploracion_nuevas] = '1'	Afectación coronaria	radio<br>1 Si<br>0 No<br>99 Desconocido<br><br>Custom alignment: RH
316	grados3_dilatacion<br>Show the field ONLY if: [afectacion3_coronaria] = '1'	Grados de dilatación coronaria<br><br>Se utilizarán como valores normales los publicados por Delaire y Dahdah (conocidos como Z-Score de Montreal) y se utilizarán los siguientes criterios:<br><br> No dilatación coronaria: Z-score < 2<br><br> Ectasia o dilatación: el máximo Z-score ≥ 2 y < 2.5<br><br> Aneurisma pequeño a moderado: Z-score ≥ 2.5 y el diámetro interno < 5 mm<br><br> Aneurisma grande: Z-score ≥ 5 y < 10 y el diámetro interno < 8 mm<br><br> Aneurisma gigante: Z-score ≥ 10 y o el diámetro interno ≥ 8 mm<br><br>Disponible una calculadora en: http://parameterz.blogspot.com.es/2010/11/montreal-coronary-artery-z-scores.html	descriptive

317	hiperrefringencia3<br>Show the field ONLY if: [afectacion3_coronaria] = '1'	Hiperrefringencia	radio<br>1 Si<br>0 No<br>99 Desconocido<br>Custom alignment: RH
318	hiperrrefringencia3_evol<br>Show the field ONLY if: [hiperrefringencia3] = '1'	Evolución	radio<br>1 Transitoria<br>2 Persistente (mas de 6-8 semanas de enfermedad)<br>3 Desconocido<br>Custom alignment: LV
319	ectasia3<br>Show the field ONLY if: [afectacion3_coronaria] = '1'	Ectasia	radio<br>1 Si<br>0 No<br>99 Desconocido<br>Custom alignment: RH
320	ectasia3_localizacion<br>Show the field ONLY if: [ectasia3] = '1'	Localización	checkbox<br>1 ectasia3_localizacion___1 Arteria coronaria principal izquierda = LCA<br>2 ectasia3_localizacion___2 Circunfleja = CX<br>3 ectasia3_localizacion___3 Descendente anterior izquierda = LAD<br>4 ectasia3_localizacion___4 Arteria coronaria derecha = RCA<br>5 ectasia3_localizacion___5 Otras (especificar)<br>Custom alignment: LV
321	ectasia3_localizacion_otra<br>Show the field ONLY if: [ectasia3_localizacion(5)] = '1'	Especificar	text
322	ectasia3_evol<br>Show the field ONLY if: [ectasia3] = '1'	Evolución	radio<br>1 Transitoria<br>2 Persistente (mas de 6-8 semanas de enfermedad)<br>3 Desconocido<br>Custom alignment: LV
323	aneurismas3<br>Show the field ONLY if: [afectacion3_coronaria] = '1'	Aneurismas	radio<br>1 Si<br>0 No<br>99 Desconocido<br>Custom alignment: RH
324	aneurismas3_numero<br>Show the field ONLY if: [aneurismas3] = '1'	Numero de aneurismas	text (integer)
325	aneurismas3_tamano<br>Show the field ONLY if: [aneurismas3] = '1'	Tamaño de aneurisma mayor<br>*mm*	text (number)
326	aneurismas3_zscore<br>Show the field ONLY if: [aneurismas3] = '1'	Z score aneurisma mayor (Clasificación Montreal)	radio<br>1 Aneurisma pequeño a moderado: Z-score ≥2.5 y el diámetro interno < 5 mm<br>2 Aneurisma grande: Z-score ≥5 y < 10 y el diámetro interno < 8 mm<br>3 Aneurisma gigante: Z-score ≥10 y el diámetro interno ≥8 mm<br>Custom alignment: LV
327	aneurismas3_vasos_afectados<br>Show the field ONLY if: [aneurismas3] = '1'	Número de vasos afectados	text (number)
328		Localización	checkbox<br>1 aneurismas3_localizacion___1

	aneurismas3_localizacion<br>Show the field ONLY if: [aneurismas3] = '1'		(continued) Arteria coronaria principal izquierda = LCA<br>2 | aneurismas3_localizacion___2 | Circunfleja = CX<br>3 | aneurismas3_localizacion___3 | Descendente anterior izquierda = LAD<br>4 | aneurismas3_localizacion___4 | Arteria coronaria derecha = RCA<br>5 | aneurismas3_localizacion___5 | Otras (especificar)<br>Custom alignment: LV
329	aneurismas3_localizacion_otra<br>Show the field ONLY if: [aneurismas3_localizacion(5)] = '1'	Especificar	text
330	aneurismas3_evol<br>Show the field ONLY if: [aneurismas3] = '1'	Evolución	radio<br>1 | Transitoria<br>2 | Persistente (mas de 6-8 semanas de enfermedad)<br>3 | Desconocido<br>Custom alignment: LV
331	aneurismas3_resul<br>Show the field ONLY if: [aneurismas3] = '1'	Resolución completa	radio<br>1 | Si<br>0 | No<br>99 | Desconocido<br>Custom alignment: RH
332	aneurismas3_semanas<br>Show the field ONLY if: [aneurismas3_resul] = '1'	Semanas desde inicio de enfermedad<br>*semanas*	text (integer)
333	estenosis3<br>Show the field ONLY if: [afectacion3_coronaria] = '1'	Estenosis	radio<br>1 | Si<br>0 | No<br>99 | Desconocido<br>Custom alignment: RH
334	estenosis3_localizacion<br>Show the field ONLY if: [estenosis3] = '1'	Localización	checkbox<br>1 | estenosis3_localizacion___1 | Arteria coronaria principal izquierda = LCA<br>2 | estenosis3_localizacion___2 | Circunfleja = CX<br>3 | estenosis3_localizacion___3 | Descendente anterior izquierda = LAD<br>4 | estenosis3_localizacion___4 | Arteria coronaria derecha = RCA<br>5 | estenosis3_localizacion___5 | Otras (especificar)<br>Custom alignment: LV
335	estenosis3_localizacion_otra<br>Show the field ONLY if: [estenosis3_localizacion(5)] = '1'	Especificar	text
336	angiotac3<br>Show the field ONLY if: [afectacion3_coronaria] = '1'	AngioTAC	radio<br>1 | Si<br>0 | No<br>99 | Desconocido

			Custom alignment: RH
337	angiotac3_semanas<br>Show the field ONLY if: [angiotac3] = 1	Semanas desde inicio de enfermedad<br>*semanas*	text (integer)
338	angiotac3_resul<br>Show the field ONLY if: [angiotac3] = 1	Resultado	notes<br>Custom alignment: LV
339	coronariografia3<br>Show the field ONLY if: [afectacion3_coronaria] = '1'	Coronariografía	radio<br>1 Si<br>0 No<br>99 Desconocido<br>Custom alignment: RH
340	coronariografia3_semanas<br>Show the field ONLY if: [coronariografia3] = 1	Semanas desde inicio de enfermedad<br>*semanas*	text (integer)
341	coronariografia3_resul<br>Show the field ONLY if: [coronariografia3] = 1	Resultado	notes<br>Custom alignment: LV
342	derrame3_pericardico<br>Show the field ONLY if: [exploracion_nuevas] = '1'	Derrame pericárdico	radio<br>1 Si<br>0 No<br>99 Desconocido<br>Custom alignment: RH
343	derrame3_pericardico_gravedad<br>Show the field ONLY if: [derrame3_pericardico] = 1	Gravedad	radio<br>1 Leve (incluye mínimo derrame e hiperrefringencia pericárdica)<br>2 Moderado<br>3 Grave<br>Custom alignment: LV
344	derrame3_pericardico_max<br>Show the field ONLY if: [derrame3_pericardico] = '1'	Máximo derrame<br>*mm*	text (number)
345	derrame3_pericardico_evol<br>Show the field ONLY if: [derrame3_pericardico] = 1	Evolución	radio<br>1 Transitoria<br>2 Persistente (mas de 6-8 semanas de enfermedad)<br>3 Desconocido<br>Custom alignment: LV
346	alteracion3_valvular<br>Show the field ONLY if: [exploracion_nuevas] = '1'	Alteración valvular	radio<br>1 Si<br>0 No<br>99 Desconocido<br>Custom alignment: RH
347	alteracion3_valvular_tipo<br>Show the field ONLY if: [alteracion3_valvular] = 1		checkbox<br>1 alteracion3_valvular_tipo___1 Insuficiencia mitral<br>2 alteracion3_valvular_tipo___2 Insuficiencia tricuspide<br>3 alteracion3_valvular_tipo___3 Insuficiencia aortica<br>4 alteracion3_valvular_tipo___4 Insuficiencia pulmonar<br>Custom alignment: LV
348	insuficiencia3_mitral_grado<br>Show the field ONLY if: [alteracion3_valvular_tipo(1)] = '1'	Insuficiencia mitral	radio (Matrix)<br>1 Leve<br>2 Moderada<br>3 Severa
349	insuficiencia3_tricuspide_grado<br>Show the field ONLY if: [alteracion3_valvular_tipo(2)] = '1'	Insuficiencia tricuspide	radio (Matrix)<br>1 Leve<br>2 Moderada<br>3 Severa

350	insuficiencia3_aortica_grado<br>Show the field ONLY if: [alteracion3_valvular_tipo(3)] = '1'	Insuficiencia aortica	radio (Matrix)<br>1 Leve<br>2 Moderada<br>3 Severa
351	insuficiencia3_pulmonar_grado<br>Show the field ONLY if: [alteracion3_valvular_tipo(4)] = '1'	Insuficiencia pulmonar	radio (Matrix)<br>1 Leve<br>2 Moderada<br>3 Severa
352	insuficiencia3_mitral_evol<br>Show the field ONLY if: [alteracion3_valvular_tipo(1)] = '1'	Insuficiencia mitral	radio (Matrix)<br>1 Transitoria<br>2 Persistente (mas de 6-8 semanas de enfermedad)<br>3 Desconocido
353	insuficiencia3_tricuspide_evol<br>Show the field ONLY if: [alteracion3_valvular_tipo(2)] = '1'	Insuficiencia tricuspide	radio (Matrix)<br>1 Transitoria<br>2 Persistente (mas de 6-8 semanas de enfermedad)<br>3 Desconocido
354	insuficiencia3_aortica_evol<br>Show the field ONLY if: [alteracion3_valvular_tipo(3)] = '1'	Insuficiencia aortica	radio (Matrix)<br>1 Transitoria<br>2 Persistente (mas de 6-8 semanas de enfermedad)<br>3 Desconocido
355	insuficiencia3_pulmonar_evol<br>Show the field ONLY if: [alteracion3_valvular_tipo(4)] = '1'	Insuficiencia pulmonar	radio (Matrix)<br>1 Transitoria<br>2 Persistente (mas de 6-8 semanas de enfermedad)<br>3 Desconocido
356	disminucion3_contractilidad<br>Show the field ONLY if: [exploracion_nuevas] = '1'	Disminución de la contractilidad	radio<br>1 Si<br>0 No<br>99 Desconocido<br><br>Custom alignment: RH
357	disfuncion3_sistolica<br>Show the field ONLY if: [disminucion3_contractilidad] = '1'	Disfunción sistólica de VI	radio<br>1 Si<br>0 No<br><br>Custom alignment: RH
358	disfuncion3_sistolica_fraccion<br>Show the field ONLY if: [disfuncion3_sistolica] = '1'	Fracción acortamiento del VI<br>%	text (number)
359	disfuncion3_sistolica_evol<br>Show the field ONLY if: [disfuncion3_sistolica] = 1	Evolución	radio<br>1 Transitoria<br>2 Persistente (mas de 6-8 semanas de enfermedad)<br>3 Desconocido<br><br>Custom alignment: LV
360	disfuncion3_segmentaria<br>Show the field ONLY if: [disminucion3_contractilidad] = '1'	Disfunción segmentaria	radio<br>1 Si<br>0 No<br><br>Custom alignment: RH
361	disfuncion3_segmentaria_localizacion<br>Show the field ONLY if: [disfuncion3_segmentaria] = '1'	Localización	checkbox<br>1 disfuncion3_segmentaria_localizacion___1 Anter (sept regio anter<br>2 disfuncion3_segmentaria_localizacion___2 Infer<br>3 disfuncion3_segmentaria_localizacion___3 Pare<br>4 disfuncion3_segmentaria_localizacion___4 Ventr derec

			Custom alignment: LV
362	disfuncion3_segmentaria_ev ol<br>Show the field ONLY if: [disf uncion3_segmentaria] = 1	Evolución	radio<br>1 Transitoria<br>2 Persistente (mas de 6-8 semanas de enfermedad)<br>3 Desconocido<br><br>Custom alignment: LV
363	exploracion_resolucion<br>Show the field ONLY if: [expl oracion_alteraciones] = '1'	Resolución de las anteriores	radio<br>1 Si<br>0 No<br>99 Desconocido<br><br>Custom alignment: RH
364	exploracion_resolucion_esp e<br>Show the field ONLY if: [expl oracion_resolucion] = '1'		checkbox<br>1 exploracion_resolucion_espe___1 Afectación coronaria<br>2 exploracion_resolucion_espe___2 Derrame pericárdico<br>3 exploracion_resolucion_espe___3 Alteraciones valvulares<br>4 exploracion_resolucion_espe___4 Disminución de la contractilidad<br><br>Custom alignment: LV
365	ecg2_realizado	Realizado	radio<br>1 Si<br>0 No<br>99 Desconocido<br><br>Custom alignment: RH
366	ecg2_escaneado<br>Show the field ONLY if: [ecg 2_realizado] = 1	Adjuntar escaneado<br>DESCARGAR COMO PDF AL MENOS UNO EN PERIODO DE RECUPERACIÓN (6-8 SEMANAS) O POSTERIOR E INDICAR LA SEMANA DE OBTENCIÓN DESDE EL INICIO DE ENFEMEDAD	file<br>Custom alignment: LV
367	ecg2_escaneado_semanas<br>Show the field ONLY if: [ecg 2_realizado] = '1'	Semanas de inicio de enfermedad<br>*semanas*	text (integer)
368	ecg2_alteraciones<br>Show the field ONLY if: [ecg 2_realizado] = '1'	Alteraciones	radio<br>1 Si<br>0 No<br>99 Desconocido<br><br>Custom alignment: RH
369	ecg2_persistencia<br>Show the field ONLY if: [ecg 2_alteraciones] = '1'	Persistencia de las lesiones anteriores	radio<br>1 Si<br>0 No<br>99 Desconocido<br><br>Custom alignment: RH
370	ecg2_persistencia_alteracio nes<br>Show the field ONLY if: [ecg 2_persistencia] = 1		radio<br>1 Bajo voltaje<br>2 Alteraciones del ritmo<br>3 Bloqueos de rama<br>4 Bloqueos de la conducción AV<br>5 Alteración de la repolarización<br><br>Custom alignment: LV
371	ecg2_persistencia_descripci on<br>Show the field ONLY if: [ecg 2_persistencia] = 1	Descripción	notes<br>Custom alignment: LV
372	ecg2_persistencia_evol<br>Show the field ONLY if: [ecg 2_persistencia] = 1	Evolución	radio<br>1 Transitoria<br>2

			Persistente (mas de 6-8 semanas de enfermedad)<br>3 Desconocido<br><br>Custom alignment: LV
373	ecg2_nuevas<br>Show the field ONLY if: [ecg 2_alteraciones] = '1'	Nuevas alteraciones	radio<br>1 Si<br>0 No<br>99 Desconocido<br><br>Custom alignment: RH
374	ecg2_nuevas_alteraciones<br>Show the field ONLY if: [ecg 2_nuevas] = 1		radio<br>1 Bajo voltaje<br>2 Alteraciones del ritmo<br>3 Bloqueos de rama<br>4 Bloqueos de la conducción AV<br>5 Alteración de la repolarización<br><br>Custom alignment: LV
375	ecg2_nuevas_descripcion<br>Show the field ONLY if: [ecg 2_nuevas] = 1	Descripción	notes<br>Custom alignment: LV
376	ecg2_nuevas_evol<br>Show the field ONLY if: [ecg 2_nuevas] = 1	Evolución	radio<br>1 Transitoria<br>2 Persistente (mas de 6-8 semanas de enfermedad)<br>3 Desconocido<br><br>Custom alignment: LV
377	ecg2_resolucion<br>Show the field ONLY if: [ecg 2_alteraciones] = '1'	Resolución de las anteriores	radio<br>1 Si<br>0 No<br>99 Desconocido<br><br>Custom alignment: RH
378	ecg2_resolucion_alteraciones<br>Show the field ONLY if: [ecg 2_resolucion] = 1		radio<br>1 Bajo voltaje<br>2 Alteraciones del ritmo<br>3 Bloqueos de rama<br>4 Bloqueos de la conducción AV<br>5 Alteración de la repolarización<br><br>Custom alignment: LV
379	comentarios2_6	Comentarios	notes<br>Custom alignment: LV
380	exploracin_cardiaca_complete	Complete?	dropdown<br>0 Incomplete<br>1 Unverified<br>2 Complete
Instrument: **Exploración cardiaca**			
381	ecocardiograma22		radio<br>1 Si<br>0 No<br>99 Desconocido<br><br>Custom alignment: RH
382	exploracion_alteraciones2<br>Show the field ONLY if: [eco cardiograma22] = '1'	Alteraciones	radio<br>1 Si<br>0 No<br>99 Desconocido<br><br>Custom alignment: RH
383		Persistencia de las lesiones anteriores	radio<br>1 Si

	exploracion_persistencia2<br>Show the field ONLY if: [exploracion_alteraciones2] = '1'		0 | No<br>99 | Desconocido<br><br>Custom alignment: RH
384	afectacion22_coronaria<br>Show the field ONLY if: [exploracion_persistencia2] = '1'	Afectación coronaria	radio<br>1 | Si<br>0 | No<br>99 | Desconocido<br><br>Custom alignment: RH
385	grados22_dilatacion<br>Show the field ONLY if: [afectacion22_coronaria] = '1'	Grados de dilatación coronaria<br><br>Se utilizarán como valores normales los publicados por Delaire y Dahdah (conocidos como Z-Score de Montreal) y se utilizarán los siguientes criterios:<br><br> No dilatación coronaria: Z-score < 2<br><br> Ectasia o dilatación: el máximo Z-score ≥ 2 y < 2.5<br><br> Aneurisma pequeño a moderado: Z-score ≥ 2.5 y el diámetro interno < 5 mm<br><br> Aneurisma grande: Z-score ≥ 5 y < 10 y el diámetro interno < 8 mm<br><br> Aneurisma gigante: Z-score ≥ 10 y o el diámetro interno ≥ 8 mm<br><br>Disponible una calculadora en: http://parameterz.blogspot.com.es/2010/11/montreal-coronary-artery-z-scores.html	descriptive
386	hiperrefringencia22<br>Show the field ONLY if: [afectacion22_coronaria] = '1'	Hiperrefringencia	radio<br>1 | Si<br>0 | No<br>99 | Desconocido<br><br>Custom alignment: RH
387	ectasia22<br>Show the field ONLY if: [afectacion22_coronaria] = '1'	Ectasia	radio<br>1 | Si<br>0 | No<br>99 | Desconocido<br><br>Custom alignment: RH
388	ectasia22_localizacion<br>Show the field ONLY if: [ectasia22] = '1'	Localización	checkbox<br>1 | ectasia22_localizacion___1 | Arteria coronaria principal izquierda = LCA<br>2 | ectasia22_localizacion___2 | Circunfleja = CX<br>3 | ectasia22_localizacion___3 | Descendente anterior izquierda = LAD<br>4 | ectasia22_localizacion___4 | Arteria coronaria derecha = RCA<br>5 | ectasia22_localizacion___5 | Otras (especificar)<br><br>Custom alignment: LV
389	ectasia22_localizacion_otra<br>Show the field ONLY if: [ectasia22_localizacion(5)] = '1'	Especificar	text
390	aneurismas22<br>Show the field ONLY if: [afectacion22_coronaria] = '1'	Aneurismas	radio<br>1 | Si<br>0 | No<br>99 | Desconocido<br><br>Custom alignment: RH
391	aneurismas22_numero<br>Show the field ONLY if: [aneurismas22] = '1'	Numero de aneurismas	text (integer)
392		Tamaño de aneurisma mayor<br>*mm*	text (number)

	aneurismas22_tamano<br>Show the field ONLY if: [aneurismas22] = '1'		
393	aneurismas22_zscore<br>Show the field ONLY if: [aneurismas22] = '1'	Z score aneurisma mayor (Clasificación Montreal)	radio<br>1 Aneurisma pequeño a moderado: Z-score ≥2.5 y el diámetro interno < 5 mm<br>2 Aneurisma grande: Z-score ≥5 y < 10 y el diámetro interno < 8 mm<br>3 Aneurisma gigante: Z-score ≥10 y el diámetro interno ≥8 mm<br>Custom alignment: LV
394	aneurismas22_vasos_afectados<br>Show the field ONLY if: [aneurismas22] = '1'	Número de vasos afectados	text (number)
395	aneurismas22_localizacion<br>Show the field ONLY if: [aneurismas22] = '1'	Localización	checkbox<br>1 aneurismas22_localizacion___1 Arteria coronaria principal izquierda = LCA<br>2 aneurismas22_localizacion___2 Circunfleja = CX<br>3 aneurismas22_localizacion___3 Descendente anterior izquierda = LAD<br>4 aneurismas22_localizacion___4 Arteria coronaria derecha = RCA<br>5 aneurismas22_localizacion___5 Otras (especificar)<br>Custom alignment: LV
396	aneurismas22_localizacion_otra<br>Show the field ONLY if: [aneurismas22_localizacion(5)] = '1'	Especificar	text
397	aneurismas22_resul<br>Show the field ONLY if: [aneurismas22] = '1'	Resolución completa	radio<br>1 Si<br>0 No<br>99 Desconocido<br>Custom alignment: RH
398	aneurismas22_semanas<br>Show the field ONLY if: [aneurismas22_resul] = '1'	Semanas desde inicio de enfermedad<br>*semanas*	text (integer)
399	estenosis22<br>Show the field ONLY if: [afectacion22_coronaria] = '1'	Estenosis	radio<br>1 Si<br>0 No<br>99 Desconocido<br>Custom alignment: RH
400	estenosis22_localizacion<br>Show the field ONLY if: [estenosis22] = '1'	Localización	checkbox<br>1 estenosis22_localizacion___1 Arteria coronaria principal izquierda = LCA<br>2 estenosis22_localizacion___2 Circunfleja = CX<br>3 estenosis22_localizacion___3 Descendente anterior izquierda = LAD<br>4 estenosis22_localizacion___4 Arteria coronaria derecha = RCA<br>5 estenosis22_localizacion___5 Otras (especificar)

			Custom alignment: LV
401	estenosis22_localizacion_otra<br>Show the field ONLY if: [estenosis22_localizacion(5)] = '1'	Especificar	text
402	angiotac22<br>Show the field ONLY if: [afectacion22_coronaria] = '1'	AngioTAC	radio<br>1 Si<br>0 No<br>99 Desconocido<br><br>Custom alignment: RH
403	angiotac22_semanas<br>Show the field ONLY if: [angiotac22] = 1	Semanas desde inicio de enfermedad<br>*semanas*	text (integer)
404	angiotac22_resul<br>Show the field ONLY if: [angiotac22] = 1	Resultado	notes<br>Custom alignment: LV
405	coronariografia22<br>Show the field ONLY if: [afectacion22_coronaria] = '1'	Coronariografía	radio<br>1 Si<br>0 No<br>99 Desconocido<br><br>Custom alignment: RH
406	coronariografia22_semanas<br>Show the field ONLY if: [coronariografia22] = 1	Semanas desde inicio de enfermedad<br>*semanas*	text (integer)
407	coronariografia22_resul<br>Show the field ONLY if: [coronariografia22] = 1	Resultado	notes<br>Custom alignment: LV
408	derrame22_pericardico<br>Show the field ONLY if: [exploracion_persistencia2] = '1'	Derrame pericárdico	radio<br>1 Si<br>0 No<br>99 Desconocido<br><br>Custom alignment: RH
409	derrame22_pericardico_gravedad<br>Show the field ONLY if: [derrame22_pericardico] = 1	Gravedad	radio<br>1 Leve (incluye mínimo derrame e hiperrefringencia pericárdica)<br>2 Moderado<br>3 Grave<br><br>Custom alignment: LV
410	derrame22_pericardico_max<br>Show the field ONLY if: [derrame22_pericardico] = '1'	Máximo derrame<br>*mm*	text (number)
411	alteracion22_valvular<br>Show the field ONLY if: [exploracion_persistencia2] = '1'	Alteración valvular	radio<br>1 Si<br>0 No<br>99 Desconocido<br><br>Custom alignment: RH
412	alteracion22_valvular_tipo<br>Show the field ONLY if: [alteracion22_valvular] = 1		checkbox<br>1 alteracion22_valvular_tipo___1 Insuficiencia mitral<br>2 alteracion22_valvular_tipo___2 Insuficiencia tricuspide<br>3 alteracion22_valvular_tipo___3 Insuficiencia aortica<br>4 alteracion22_valvular_tipo___4 Insuficiencia pulmonar<br><br>Custom alignment: LV
413	insuficiencia22_mitral_grado<br>Show the field ONLY if: [alteracion22_valvular_tipo(1)] = '1'	Insuficiencia mitral	radio (Matrix)<br>1 Leve<br>2 Moderada<br>3 Severa
414	insuficiencia22_tricuspide_grado	Insuficiencia tricuspide	radio (Matrix)

	Show the field ONLY if: [alteracion22_valvular_tipo(2)] = '1'		1 Leve<br>2 Moderada<br>3 Severa
415	insuficiencia22_aortica_grado<br>Show the field ONLY if: [alteracion22_valvular_tipo(3)] = '1'	Insuficiencia aortica	radio (Matrix)<br>1 Leve<br>2 Moderada<br>3 Severa
416	insuficiencia22_pulmonar_grado<br>Show the field ONLY if: [alteracion22_valvular_tipo(4)] = '1'	Insuficiencia pulmonar	radio (Matrix)<br>1 Leve<br>2 Moderada<br>3 Severa
417	disminucion22_contractilidad<br>Show the field ONLY if: [exploracion_persistencia2] = '1'	Disminución de la contractilidad	radio<br>1 Si<br>0 No<br>99 Desconocido<br><br>Custom alignment: RH
418	disfuncion22_sistolica<br>Show the field ONLY if: [disminucion22_contractilidad] = '1'	Disfunción sistólica de VI	radio<br>1 Si<br>0 No<br><br>Custom alignment: RH
419	disfuncion22_sistolica_fraccion<br>Show the field ONLY if: [disfuncion22_sistolica] = '1'	Fracción acortamiento del VI<br>%	text (number)
420	disfuncion22_segmentaria<br>Show the field ONLY if: [disminucion22_contractilidad] = '1'	Disfunción segmentaria	radio<br>1 Si<br>0 No<br><br>Custom alignment: RH
421	disfuncion22_segmentaria_localizacion<br>Show the field ONLY if: [disfuncion22_segmentaria] = '1'	Localización	radio<br>1 Anteroapical (septo, apex y regiones anteroseptales)<br>2 Inferobasal<br>3 Pared lateral<br>4 Ventriculo derecho<br><br>Custom alignment: LV
422	exploracion_nuevas2<br>Show the field ONLY if: [exploracion_alteraciones2] = '1'	Nuevas alteraciones	radio<br>1 Si<br>0 No<br>99 Desconocido<br><br>Custom alignment: RH
423	afectacion33_coronaria<br>Show the field ONLY if: [exploracion_nuevas2] = '1'	Afectación coronaria	radio<br>1 Si<br>0 No<br>99 Desconocido<br><br>Custom alignment: RH
424	grados33_dilatacion<br>Show the field ONLY if: [afectacion33_coronaria] = '1'	Grados de dilatación coronaria<br><br>Se utilizarán como valores normales los publicados por Delaire y Dahdah (conocidos como Z-Score de Montreal) y se utilizarán los siguientes criterios:<br><br> No dilatación coronaria: Z-score < 2<br><br> Ectasia o dilatación: el máximo Z-score ≥ 2 y < 2.5<br><br> Aneurisma pequeño a moderado: Z-score ≥ 2.5 y el diámetro interno < 5 mm<br><br> Aneurisma grande: Z-score ≥ 5 y < 10 y el diámetro interno < 8 mm	descriptive

		 Aneurisma gigante: Z-score ≥ 10 y o el diámetro interno ≥ 8 mm<br><br>Disponible una calculadora en: http://parameterz.blogspot.com.es/2010/11/montreal-coronary-artery-z-scores.html	
425	hiperrefringencia33<br>Show the field ONLY if: [afectacion33_coronaria] = '1'	Hiperrefringencia	radio<br>1 Si<br>0 No<br>99 Desconocido<br><br>Custom alignment: RH
426	ectasia33<br>Show the field ONLY if: [afectacion33_coronaria] = '1'	Ectasia	radio<br>1 Si<br>0 No<br>99 Desconocido<br><br>Custom alignment: RH
427	ectasia33_localizacion<br>Show the field ONLY if: [ectasia33] = '1'	Localización	checkbox<br>1 ectasia33_localizacion___1 Arteria coronaria principal izquierda = LCA<br>2 ectasia33_localizacion___2 Circunfleja = CX<br>3 ectasia33_localizacion___3 Descendente anterior izquierda = LAD<br>4 ectasia33_localizacion___4 Arteria coronaria derecha = RCA<br>5 ectasia33_localizacion___5 Otras (especificar)<br><br>Custom alignment: LV
428	ectasia33_localizacion_otra<br>Show the field ONLY if: [ectasia33_localizacion(5)] = '1'	Especificar	text
429	aneurismas33<br>Show the field ONLY if: [afectacion33_coronaria] = '1'	Aneurismas	radio<br>1 Si<br>0 No<br>99 Desconocido<br><br>Custom alignment: RH
430	aneurismas33_numero<br>Show the field ONLY if: [aneurismas33] = '1'	Numero de aneurismas	text (integer)
431	aneurismas33_tamano<br>Show the field ONLY if: [aneurismas33] = '1'	Tamaño de aneurisma mayor<br>*mm*	text (number)
432	aneurismas33_zscore<br>Show the field ONLY if: [aneurismas33] = '1'	Z score aneurisma mayor (Clasificación Montreal)	radio<br>1 Aneurisma pequeño a moderado: Z-score ≥2.5 y el diámetro interno < 5 mm<br>2 Aneurisma grande: Z-score ≥5 y < 10 y el diámetro interno < 8 mm<br>3 Aneurisma gigante: Z-score ≥10 y el diámetro interno ≥8 mm<br><br>Custom alignment: LV
433	aneurismas33_vasos_afectados<br>Show the field ONLY if: [aneurismas33] = '1'	Número de vasos afectados	text (number)
434	aneurismas33_localizacion<br>Show the field ONLY if: [aneurismas33] = '1'	Localización	checkbox<br>1 aneurismas33_localizacion___1 Arteria coronaria principal izquierda = LCA<br>2 aneurismas33_localizacion___2 Circunfleja = CX<br>3 aneurismas33_localizacion___3 Descendente anterior izquierda = LAD

			4 aneurismas33_localizacion___4 Arteria coronaria derecha = RCA<br>5 aneurismas33_localizacion___5 Otras (especificar)<br>Custom alignment: LV
435	aneurismas33_localizacion_otra<br>Show the field ONLY if: [aneurismas33_localizacion(5)] = '1'	Especificar	text
436	aneurismas33_resul<br>Show the field ONLY if: [aneurismas33] = '1'	Resolución completa	radio<br>1 Si<br>0 No<br>99 Desconocido<br>Custom alignment: RH
437	aneurismas33_semanas<br>Show the field ONLY if: [aneurismas33_resul] = '1'	Semanas desde inicio de enfermedad<br>*semanas*	text (integer)
438	estenosis33<br>Show the field ONLY if: [afectacion33_coronaria] = '1'	Estenosis	radio<br>1 Si<br>0 No<br>99 Desconocido<br>Custom alignment: RH
439	estenosis33_localizacion<br>Show the field ONLY if: [estenosis33] = '1'	Localización	checkbox<br>1 estenosis33_localizacion___1 Arteria coronaria principal izquierda = LCA<br>2 estenosis33_localizacion___2 Circunfleja = CX<br>3 estenosis33_localizacion___3 Descendente anterior izquierda = LAD<br>4 estenosis33_localizacion___4 Arteria coronaria derecha = RCA<br>5 estenosis33_localizacion___5 Otras (especificar)<br>Custom alignment: LV
440	estenosis33_localizacion_otra<br>Show the field ONLY if: [estenosis33_localizacion(5)] = '1'	Especificar	text
441	angiotac33<br>Show the field ONLY if: [afectacion33_coronaria] = '1'	AngioTAC	radio<br>1 Si<br>0 No<br>99 Desconocido<br>Custom alignment: RH
442	angiotac33_semanas<br>Show the field ONLY if: [angiotac33] = 1	Semanas desde inicio de enfermedad<br>*semanas*	text (integer)
443	angiotac33_resul<br>Show the field ONLY if: [angiotac33] = 1	Resultado	notes<br>Custom alignment: LV
444	coronariografia33<br>Show the field ONLY if: [afectacion33_coronaria] = '1'	Coronariografía	radio<br>1 Si<br>0 No<br>99 Desconocido<br>Custom alignment: RH
445	coronariografia33_semanas<br>Show the field ONLY if: [coronariografia33] = 1	Semanas desde inicio de enfermedad<br>*semanas*	text (integer)

446	coronariografia33_resul<br>Show the field ONLY if: [coronariografia33] = 1	Resultado	notes<br>Custom alignment: LV
447	derrame33_pericardico<br>Show the field ONLY if: [exploracion_nuevas2] = '1'	Derrame pericárdico	radio<br>1 Si<br>0 No<br>99 Desconocido<br>Custom alignment: RH
448	derrame33_pericardico_gravedad<br>Show the field ONLY if: [derrame33_pericardico] = 1	Gravedad	radio<br>1 Leve (incluye mínimo derrame e hiperrefringencia pericárdica)<br>2 Moderado<br>3 Grave<br>Custom alignment: LV
449	derrame33_pericardico_max<br>Show the field ONLY if: [derrame33_pericardico] = '1'	Máximo derrame<br>*mm*	text (number)
450	alteracion33_valvular<br>Show the field ONLY if: [exploracion_nuevas2] = '1'	Alteración valvular	radio<br>1 Si<br>0 No<br>99 Desconocido<br>Custom alignment: RH
451	alteracion33_valvular_tipo<br>Show the field ONLY if: [alteracion33_valvular] = 1		checkbox<br>1 alteracion33_valvular_tipo___1 Insuficiencia mitral<br>2 alteracion33_valvular_tipo___2 Insuficiencia tricuspide<br>3 alteracion33_valvular_tipo___3 Insuficiencia aortica<br>4 alteracion33_valvular_tipo___4 Insuficiencia pulmonar<br>Custom alignment: LV
452	insuficiencia33_mitral_grado<br>Show the field ONLY if: [alteracion33_valvular_tipo(1)] = '1'	Insuficiencia mitral	radio (Matrix)<br>1 Leve<br>2 Moderada<br>3 Severa
453	insuficiencia33_tricuspide_grado<br>Show the field ONLY if: [alteracion33_valvular_tipo(2)] = '1'	Insuficiencia tricuspide	radio (Matrix)<br>1 Leve<br>2 Moderada<br>3 Severa
454	insuficiencia33_aortica_grado<br>Show the field ONLY if: [alteracion33_valvular_tipo(3)] = '1'	Insuficiencia aortica	radio (Matrix)<br>1 Leve<br>2 Moderada<br>3 Severa
455	insuficiencia33_pulmonar_grado<br>Show the field ONLY if: [alteracion33_valvular_tipo(4)] = '1'	Insuficiencia pulmonar	radio (Matrix)<br>1 Leve<br>2 Moderada<br>3 Severa
456	disminucion33_contractilidad<br>Show the field ONLY if: [exploracion_nuevas2] = '1'	Disminución de la contractilidad	radio<br>1 Si<br>0 No<br>99 Desconocido<br>Custom alignment: RH
457	disfuncion33_sistolica<br>Show the field ONLY if: [disminucion33_contractilidad] = '1'	Disfunción sistólica de VI	radio<br>1 Si<br>0 No<br>Custom alignment: RH
458	disfuncion33_sistolica_fraccion	Fracción acortamiento del VI<br>*%*	text (number)

	Show the field ONLY if: [disfuncion33_sistolica] = '1'		
459	disfuncion33_segmentaria<br>Show the field ONLY if: [disminucion33_contractilidad] = '1'	Disfunción segmentaria	radio<br>1 Si<br>0 No<br>Custom alignment: RH
460	disfuncion33_segmentaria_localizacion<br>Show the field ONLY if: [disfuncion33_segmentaria] = '1'	Localización	checkbox<br>1 disfuncion33_segmentaria_localizacion___1 Ante[illegible] (sep[illegible] regi[illegible] ante[illegible]<br>2 disfuncion33_segmentaria_localizacion___2 Infe[illegible]<br>3 disfuncion33_segmentaria_localizacion___3 Pare[illegible]<br>4 disfuncion33_segmentaria_localizacion___4 Ven[illegible] dere[illegible]<br>Custom alignment: LV
461	exploracion_resolucion2<br>Show the field ONLY if: [exploracion_alteraciones2] = '1'	Resolución de las anteriores	radio<br>1 Si<br>0 No<br>99 Desconocido<br>Custom alignment: RH
462	exploracion_resolucion_espe2<br>Show the field ONLY if: [exploracion_resolucion2] = '1'		checkbox<br>1 exploracion_resolucion_espe2___1 Afectación coronaria<br>2 exploracion_resolucion_espe2___2 Derrame pericárdico<br>3 exploracion_resolucion_espe2___3 Alteraciones valvulares<br>4 exploracion_resolucion_espe2___4 Disminución de la contractilidad[illegible]<br>Custom alignment: LV
463	ecg22_realizado	Realizado	radio<br>1 Si<br>0 No<br>99 Desconocido<br>Custom alignment: RH
464	ecg22_escaneado<br>Show the field ONLY if: [ecg22_realizado] = 1	Adjuntar escaneado<br>DESCARGAR COMO PDF AL MENOS UNO EN PERIODO DE RECUPERACIÓN (6-8 SEMANAS) O POSTERIOR E INDICAR LA SEMANA DE OBTENCIÓN DESDE EL INICIO DE ENFEMEDAD	file<br>Custom alignment: LV
465	ecg22_escaneado_semanas<br>Show the field ONLY if: [ecg22_realizado] = '1'	Semanas de inicio de enfermedad<br>*semanas*	text (integer)
466	ecg22_alteraciones<br>Show the field ONLY if: [ecg22_realizado] = '1'	Alteraciones	radio<br>1 Si<br>0 No<br>99 Desconocido<br>Custom alignment: RH
467	ecg22_persistencia<br>Show the field ONLY if: [ecg22_alteraciones] = '1'	Persistencia de las lesiones anteriores	radio<br>1 Si<br>0 No<br>99 Desconocido<br>Custom alignment: RH
468	ecg22_persistencia_alteraciones<br>Show the field ONLY if: [ecg22_persistencia] = 1		radio<br>1 Bajo voltaje<br>2 Alteraciones del ritmo<br>3 Bloqueos de rama<br>4 Bloqueos de la conducción AV<br>5 Alteración de la repolarización

			Custom alignment: LV
469	ecg22_persistencia_descripcion<br>Show the field ONLY if: [ecg22_persistencia] = 1	Descripción	notes<br>Custom alignment: LV
470	ecg22_nuevas<br>Show the field ONLY if: [ecg22_alteraciones] = '1'	Nuevas alteraciones	radio<br>1 Si<br>0 No<br>99 Desconocido<br>Custom alignment: RH
471	ecg22_nuevas_alteraciones<br>Show the field ONLY if: [ecg22_nuevas] = 1		radio<br>1 Bajo voltaje<br>2 Alteraciones del ritmo<br>3 Bloqueos de rama<br>4 Bloqueos de la conducción AV<br>5 Alteración de la repolarización<br>Custom alignment: LV
472	ecg22_nuevas_descripcion<br>Show the field ONLY if: [ecg22_nuevas] = 1	Descripción	notes<br>Custom alignment: LV
473	ecg22_resolucion<br>Show the field ONLY if: [ecg22_alteraciones] = '1'	Resolución de las anteriores	radio<br>1 Si<br>0 No<br>99 Desconocido<br>Custom alignment: RH
474	ecg22_resolucion_alteraciones<br>Show the field ONLY if: [ecg22_resolucion] = 1		radio<br>1 Bajo voltaje<br>2 Alteraciones del ritmo<br>3 Bloqueos de rama<br>4 Bloqueos de la conducción AV<br>5 Alteración de la repolarización<br>Custom alignment: LV
475	comentarios22_6	Comentarios	notes<br>Custom alignment: LV
476	exploracin_cardiaca_5cf8_complete	Complete?	dropdown<br>0 Incomplete<br>1 Unverified<br>2 Complete
Instrument: **Otras pruebas complementarias**			
477	rx_torax	Radiografía de tórax	radio<br>1 Si<br>0 No<br>Custom alignment: RH
478	rx_torax_resultado<br>Show the field ONLY if: [rx_torax] = '1'	Resultado	radio<br>1 Normal<br>2 Anormal<br>Custom alignment: RH
479	rx_torax_anormal<br>Show the field ONLY if: [rx_torax_resultado] = '2'	Especificar	radio<br>1 Neumonía/infiltrado intersticial<br>2 Engrosamientos peribronquiales<br>3 Adenopatías mediastínicas<br>4 Atelectasia<br>5 Derrame pleural<br>6 Otros (especificar)<br>Custom alignment: LV
480		Especificar	text

	rx_torax_anormal_espe<br>Show the field ONLY if: [rx_torax_anormal] = '6'		
481	test_antigenico	Test rápido antigénico SGA (estreptococo del grupo A/pyogenes)	radio<br>1 Si<br>0 No<br>Custom alignment: RH
482	test_antigenico_resultado<br>Show the field ONLY if: [test_antigenico] = '1'	Resultado	radio<br>0 Negativo<br>1 Positivo<br>Custom alignment: RH
483	frotis_faringeo	Frotis faríngeo para bacterias	radio<br>1 Si<br>0 No<br>Custom alignment: RH
484	frotis_faringeo_resultado<br>Show the field ONLY if: [frotis_faringeo] = '1'	Resultado	radio<br>1 SGA (estreptococo del grupo A/pyogenes)<br>2 Flora saprófita<br>3 Cándida sp<br>4 Otros (especificar)<br>Custom alignment: LV
485	frotis_faringeo_resul_otro<br>Show the field ONLY if: [frotis_faringeo_resultado] = '4'	Especificar	text
486	frotis_nasofaringeo	Frotis/aspirado nasofaríngeo para virus	radio<br>1 Si<br>0 No<br>Custom alignment: RH
487	frotis_nasofaringeo_resul<br>Show the field ONLY if: [frotis_nasofaringeo] = 1	Resultado	radio<br>0 Negativo<br>1 Positivo<br>Custom alignment: RH
488	frotis_nasofaringeo_espe<br>Show the field ONLY if: [frotis_nasofaringeo_resul] = '1'	Especificar (se puede marcar mas de uno)	checkbox<br>1 frotis_nasofaringeo_espe___1 Gripe<br>2 frotis_nasofaringeo_espe___2 VRS<br>3 frotis_nasofaringeo_espe___3 Parainfluenza<br>4 frotis_nasofaringeo_espe___4 Metaneumovirus<br>5 frotis_nasofaringeo_espe___5 Rinovirus<br>6 frotis_nasofaringeo_espe___6 Adenovirus<br>7 frotis_nasofaringeo_espe___7 Bocavirus<br>8 frotis_nasofaringeo_espe___8 Otros (especificar)<br>Custom alignment: LV
489	frotis_nasofaringeo_otro<br>Show the field ONLY if: [frotis_nasofaringeo_espe(8)] = '1'	Especificar	text
490	tecnica_gripe<br>Show the field ONLY if: [frotis_nasofaringeo_espe(1)] = '1'	Gripe	radio (Matrix)<br>1 Test rápido/antigénico<br>2 Cultivo<br>3 PCR
491	tecnica_vrs<br>Show the field ONLY if: [frotis_nasofaringeo_espe(2)] = '1'	VRS	radio (Matrix)<br>1 Test rápido/antigénico<br>2 Cultivo<br>3 PCR
492	tecnica_parainfluenza<br>Show the field ONLY if: [frotis_nasofaringeo_espe(3)] = '1'	Parainfluenza	radio (Matrix)<br>1 Test rápido/antigénico<br>2 Cultivo

			3 PCR
493	tecnica_metaneumovirus<br>Show the field ONLY if: [frotis_nasofaringeo_espe(4)] = '1'	Metaneumovirus	radio (Matrix)<br>1 Test rápido/antigénico<br>2 Cultivo<br>3 PCR
494	tecnica_rinovirus<br>Show the field ONLY if: [frotis_nasofaringeo_espe(5)] = '1'	Rinovirus	radio (Matrix)<br>1 Test rápido/antigénico<br>2 Cultivo<br>3 PCR
495	tecnica_adenovirus<br>Show the field ONLY if: [frotis_nasofaringeo_espe(6)] = '1'	Adenovirus	radio (Matrix)<br>1 Test rápido/antigénico<br>2 Cultivo<br>3 PCR
496	tecnica_bocavirus<br>Show the field ONLY if: [frotis_nasofaringeo_espe(7)] = '1'	Bocavirus	radio (Matrix)<br>1 Test rápido/antigénico<br>2 Cultivo<br>3 PCR
497	tecnica_otros<br>Show the field ONLY if: [frotis_nasofaringeo_espe(8)] = '1'	Otros	radio (Matrix)<br>1 Test rápido/antigénico<br>2 Cultivo<br>3 PCR
498	hemocultivo	Hemocultivo	radio<br>1 Si<br>0 No<br>Custom alignment: RH
499	hemocultivo_resultado<br>Show the field ONLY if: [hemocultivo] = '1'	Resultado	checkbox<br>1 hemocultivo_resultado___1 Estéril<br>2 hemocultivo_resultado___2 S. pyogenes<br>3 hemocultivo_resultado___3 S. aureus<br>4 hemocultivo_resultado___4 Neumococo<br>5 hemocultivo_resultado___5 M. catarrhalis<br>6 hemocultivo_resultado___6 H. influenza<br>7 hemocultivo_resultado___7 Enterococo<br>8 hemocultivo_resultado___8 E. coli<br>9 hemocultivo_resultado___9 Otro BGN (bacilo gram negativo)<br>10 hemocultivo_resultado___10 Candida sp<br>11 hemocultivo_resultado___11 Otros (especificar)<br>Custom alignment: LV
500	hemocultivo_otro<br>Show the field ONLY if: [hemocultivo_resultado(11)] = '1'	Especificar	text
501	coprocultivo	Coprocultivo	radio<br>1 Si<br>0 No<br>Custom alignment: RH
502	coprocultivo_resultado<br>Show the field ONLY if: [coprocultivo] = '1'	Resultado (se puede marcar mas de uno)	checkbox<br>1 coprocultivo_resultado___1 Negativo<br>2 coprocultivo_resultado___2 Rotavirus<br>3 coprocultivo_resultado___3 Adenovirus<br>4 coprocultivo_resultado___4 Otro virus<br>5 coprocultivo_resultado___5 Salmonella<br>6 coprocultivo_resultado___6 Campylobacter<br>7 coprocultivo_resultado___7 Yersinia<br>8 coprocultivo_resultado___8

			E coli enteroxigenico/ent<br>9 coprocultivo_resultado___9 Clostridium dificile<br>10 coprocultivo_resultado___10 Otras bacterias enteropatógenas<br>Custom alignment: LV
503	coprocultivo_otro<br>Show the field ONLY if: [coprocultivo_resultado(10)] = '1'	Especificar	text
504	urocultivo	Urocultivo	radio<br>1 Si<br>0 No<br>Custom alignment: RH
505	urocultivo_resultado<br>Show the field ONLY if: [urocultivo] = '1'	Resultado	checkbox<br>1 urocultivo_resultado___1 Esteril<br>2 urocultivo_resultado___2 E.coli<br>3 urocultivo_resultado___3 Proteus mirabilis<br>4 urocultivo_resultado___4 Psudomonas<br>5 urocultivo_resultado___5 Klebsiella<br>6 urocultivo_resultado___6 Enterococo<br>7 urocultivo_resultado___7 Otros (especificar)<br>Custom alignment: LV
506	urocultivo_otro<br>Show the field ONLY if: [urocultivo_resultado(7)] = '1'	Especificar	text
507	fondo_ojo	Fondo de ojo	radio<br>1 Si<br>0 No<br>Custom alignment: RH
508	fondo_ojo_resultado<br>Show the field ONLY if: [fondo_ojo] = '1'	Resultado	radio<br>1 Normal<br>2 Alterado (especificar)<br>Custom alignment: RH
509	fondo_ojo_espe<br>Show the field ONLY if: [fondo_ojo_resultado] = '2'	Especificar	text
510	eco_abdominal	Eco abdominal	radio<br>1 Si<br>0 No<br>Custom alignment: RH
511	eco_abdominal_resul<br>Show the field ONLY if: [eco_abdominal] = '1'	Resultado	radio<br>1 Normal<br>2 Hepatomegalia<br>3 Esplenomegalia<br>4 Adenitis mesentérica<br>5 Ascitis<br>6 Hidrops vesicular<br>7 Otros (especificar)<br>Custom alignment: LV
512	eco_abdominal_otro<br>Show the field ONLY if: [eco_abdominal_resul] = '7'	Especificar	text
513	puncion_lumbar	Punción lumbar	radio<br>1 Si<br>0 No<br>Custom alignment: RH
514	puncion_lumbar_resul<br>Show the field ONLY if: [puncion_lumbar] = '1'	Resultado	radio<br>1 Normal

			2 Alterado (especificar)<br><br>Custom alignment: RH
515	puncion_lumbar_espe<br>Show the field ONLY if: [puncion_lumbar_resul] = '2'	Especificar	text
516	comentarios_7	Comentarios	notes<br>Custom alignment: LV
517	otras_pruebas_complementarias_complete	Complete?	dropdown<br>0 Incomplete<br>1 Unverified<br>2 Complete
Instrument: **Tratamiento**			
518	igiv	IGIV (gammaglobulina intravenosa)	radio<br>1 Si<br>0 No<br><br>Custom alignment: RH
519	igiv_dosis<br>Show the field ONLY if: [igiv] = '1'	Dosis	radio<br>1 2 gr/kg<br>2 Otras (especificar en gr/kg)<br><br>Custom alignment: RH
520	igiv_dosis_espe<br>Show the field ONLY if: [igiv_dosis] = '2'	Especificar	text
521	igiv_marca<br>Show the field ONLY if: [igiv] = '1'	Marca comercial	text
522	igiv_fecha_dosis<br>Show the field ONLY if: [igiv] = '1'	Fecha primera dosis<br>*(dd/mm/aaaa)*	text (date_dmy)
523	efectos_adversos<br>Show the field ONLY if: [igiv] = '1'	¿Efectos adversos?	radio<br>1 Si<br>0 No<br><br>Custom alignment: RH
524	efectos_adversos_espe<br>Show the field ONLY if: [efectos_adversos] = '1'	Especificar	checkbox<br>1 efectos_adversos_espe___1 Escalofrios<br>2 efectos_adversos_espe___2 Hipotensión<br>3 efectos_adversos_espe___3 "Flushing"-rubefa<br>4 efectos_adversos_espe___4 Gastrointestinale (náuseas/vómito<br>5 efectos_adversos_espe___5 Exantema<br>6 efectos_adversos_espe___6 Dolor muscular/artralgi<br>7 efectos_adversos_espe___7 Cefalea<br>8 efectos_adversos_espe___8 Mareos<br>9 efectos_adversos_espe___9 Otros (especifica<br>99 efectos_adversos_espe___99 Desconocido (Ma si en el estudio retrospectivo no e documentado en historia clínica)<br><br>Custom alignment: LV
525	efectos_adversos_otro<br>Show the field ONLY if: [efectos_adversos_espe(9)] = '1'	Especificar	text
526	rep_segunda_dosis<br>Show the field ONLY if: [igiv] = '1'	¿Se ha repetido una segunda dosis de IVIG?	radio<br>1 Si<br>0 No<br><br>Custom alignment: RH
527	segunda_dosis_horas<br>Show the field ONLY if: [rep_segunda_dosis] = '1'	¿Cuántas horas después del inicio de la infusión?<br>*horas*	text (integer)<br>Custom alignment: RH
528		Dosis	radio

	segunda_dosis<br>Show the field ONLY if: [rep_segunda_dosis] = 1		1 2 gr/kg<br>2 Otras (especificar en gr/kg)<br>Custom alignment: RH
529	segunda_dosis_espe<br>Show the field ONLY if: [segunda_dosis] = '2'	Especificar	text
530	segunda_dosis_marca<br>Show the field ONLY if: [rep_segunda_dosis] = '1'	Marca comercial	text
531	segunda_dosis_efectos_adversos<br>Show the field ONLY if: [rep_segunda_dosis] = '1'	¿Efectos adversos?	radio<br>1 Si<br>0 No<br>Custom alignment: RH
532	segunda_dosis_efectos_adversos_espe<br>Show the field ONLY if: [segunda_dosis_efectos_adversos] = '1'	Especificar	checkbox<br>1 segunda_dosis_efectos_adversos_espe___1 [illegible]<br>2 segunda_dosis_efectos_adversos_espe___2 [illegible]<br>3 segunda_dosis_efectos_adversos_espe___3 [illegible]<br>4 segunda_dosis_efectos_adversos_espe___4 [illegible]<br>5 segunda_dosis_efectos_adversos_espe___5 [illegible]<br>6 segunda_dosis_efectos_adversos_espe___6 [illegible]<br>7 segunda_dosis_efectos_adversos_espe___7 [illegible]<br>8 segunda_dosis_efectos_adversos_espe___8 [illegible]<br>9 segunda_dosis_efectos_adversos_espe___9 [illegible]<br>99 segunda_dosis_efectos_adversos_espe___99 [illegible]<br>Custom alignment: LV
533	segunda_dosis_efectos_adversos_otro<br>Show the field ONLY if: [segunda_dosis_efectos_adversos_espe(9)] = '1'	Especificar	text
534	rep_tercera_dosis<br>Show the field ONLY if: [rep_segunda_dosis] = '1'	¿Se ha repetido una tercera dosis de IVIG?	radio<br>1 Si<br>0 No<br>Custom alignment: RH
535	tercera_dosis_horas<br>Show the field ONLY if: [rep_tercera_dosis] = '1'	¿Cuántas horas después del inicio de la infusión?<br>*horas*	text (integer)<br>Custom alignment: RH
536	tercera_dosis<br>Show the field ONLY if: [rep_tercera_dosis] = 1	Dosis	radio<br>1 2 gr/kg<br>2 Otras (especificar en gr/kg)<br>Custom alignment: RH
537	tercera_dosis_espe<br>Show the field ONLY if: [tercera_dosis] = '2'	Especificar	text
538	tercera_dosis_marca<br>Show the field ONLY if: [rep_tercera_dosis] = '1'	Marca comercial	text
539	tercera_dosis_efectos_adversos<br>Show the field ONLY if: [rep_tercera_dosis] = '1'	¿Efectos adversos?	radio<br>1 Si<br>0 No<br>Custom alignment: RH
540	tercera_dosis_efectos_adversos_espe<br>Show the field ONLY if: [tercera_dosis_efectos_adversos] = '1'	Especificar	checkbox<br>1 tercera_dosis_efectos_adversos_espe___1 [illegible]<br>2 tercera_dosis_efectos_adversos_espe___2 [illegible]<br>3 tercera_dosis_efectos_adversos_espe___3 [illegible]<br>4 tercera_dosis_efectos_adversos_espe___4 [illegible]

			5 | tercera_dosis_efectos_adversos_espe___5 | [illegible]<br>6 | tercera_dosis_efectos_adversos_espe___6 | [illegible]<br>7 | tercera_dosis_efectos_adversos_espe___7 | [illegible]<br>8 | tercera_dosis_efectos_adversos_espe___8 | [illegible]<br>9 | tercera_dosis_efectos_adversos_espe___9 | [illegible]<br>99 | tercera_dosis_efectos_adversos_espe___99 | [illegible]<br><br>Custom alignment: LV
541	tercera_dosis_efectos_adversos_otro<br>Show the field ONLY if: [tercera_dosis_efectos_adversos_espe(9)] = '1'	Especificar	text
542	admin_antiinflamatorios	¿Se ha administrado antiinflamatorios en fase aguda?	radio<br>1 | Si<br>0 | No<br><br>Custom alignment: RH
543	admin_antiinflamatorios_espe<br>Show the field ONLY if: [admin_antiinflamatorios] = '1'	Especificar	radio<br>1 | AAS 30-50 mg/kg/día<br>2 | AAS 80-100 mg/kg/día<br>3 | Ibuprofeno<br>4 | Otros (especificar)<br><br>Custom alignment: LV
544	admin_antiinflamatorios_otros<br>Show the field ONLY if: [admin_antiinflamatorios_espe] = '4'	Especificar	text
545	admin_antiinflamatorios_dosis<br>Show the field ONLY if: [admin_antiinflamatorios] = '1'	Dosis<br>*mg/kg/dia*	text (number)
546	admin_antiinflamatorios_fecha<br>Show the field ONLY if: [admin_antiinflamatorios] = '1'	Fecha inicio<br>*(dd/mm/aaaa)*	text (date_dmy)
547	admin_antiinflamatorios_dias<br>Show the field ONLY if: [admin_antiinflamatorios] = '1'	Número total de días antiinflamatorios dosis alta<br>*días*	text (integer)
548	glucocorticoides	¿Ha recibido glucocorticoides?	radio<br>1 | Si<br>0 | No<br><br>Custom alignment: RH
549	choques_metilprednisolona<br>Show the field ONLY if: [glucocorticoides] = '1'	¿Ha recibido "choques" de Metilprednisolona IV?	radio<br>1 | Si<br>0 | No<br><br>Custom alignment: RH
550	choques_metilprednisolona_fecha<br>Show the field ONLY if: [choques_metilprednisolona] = '1'	Fecha inicio<br>*(dd/mm/aaaa)*	text (date_dmy)
551	choques_metilprednisolona_dosis<br>Show the field ONLY if: [choques_metilprednisolona] = '1'	Dosis<br>*mg/kg/día*	text (number)
552	choques_metilprednisolona_dias<br>Show the field ONLY if: [choques_metilprednisolona] = '1'	Número total de días<br>*días*	text (integer)
553		¿Ha recibido glucocorticoides a dosis habituales?	radio

	glucocorticoides_habituales<br>Show the field ONLY if: [glucocorticoides] = '1'		1 Si<br>0 No<br><br>Custom alignment: RH
554	glucocorticoides_razon<br>Show the field ONLY if: [glucocorticoides_habituales] = 1		radio<br>1 Tras "choques" de metilprednisolona<br>2 Inició a dosis habituales<br><br>Custom alignment: LV
555	glucocorticoides_via<br>Show the field ONLY if: [glucocorticoides_habituales] = 1	Inició el tratamiento de glucocorticoides a dosis habituales por vía	radio<br>1 Oral<br>2 I.V.<br><br>Custom alignment: RH
556	glucocorticoides_fecha<br>Show the field ONLY if: [glucocorticoides_habituales] = '1'	Fecha inicio glucocorticoides a dosis habituales<br>*(dd/mm/aaaa)*	text (date_dmy)
557	glucocorticoides_farmaco<br>Show the field ONLY if: [glucocorticoides_habituales] = '1'	Principio activo	radio<br>1 Metilprednisolona<br>2 Prednisolona<br>3 Prednisona<br>4 Dexametasona<br>5 Otro<br><br>Custom alignment: LV
558	glucocorticoides_dosis<br>Show the field ONLY if: [glucocorticoides_habituales] = '1'	Dosis inicial de glucocorticoides a dosis habituales<br>*mg/kg/dia*	text (number)
559	glucocorticoides_dias<br>Show the field ONLY if: [glucocorticoides_habituales] = '1'	Número total de días de glucocorticoides (incluyendo descenso de dosis)<br>*días*	text (integer)
560	antibioticos	¿Ha precisado antibióticos?	radio<br>1 Si<br>0 No<br><br>Custom alignment: RH
561	antibioticos_fecha<br>Show the field ONLY if: [antibioticos] = '1'	Fecha inicio<br>*(dd/mm/aaaa)*	text (date_dmy)
562	antibioticos_farmaco<br>Show the field ONLY if: [antibioticos] = '1'	Antibiótico	text
563	antibioticos_dias<br>Show the field ONLY if: [antibioticos] = '1'	Número de días<br>*días*	text (integer)
564	antiagregacion	Pauta de tratamiento antiagregante	radio<br>1 Si<br>0 No<br><br>Custom alignment: RH
565	antiagregante_tipo<br>Show the field ONLY if: [antiagregacion] = '1'	Antiagregante	radio<br>1 AAS a dosis bajas (3-5 mg/kg/dia)<br>2 Otro (especificar)
566	antiagregante_otro<br>Show the field ONLY if: [antiagregante_tipo] = '2'	Especificar	text
567	antiagregacion_finicio<br>Show the field ONLY if: [antiagregacion] = '1'	Fecha inicio<br>*(dd/mm/aaaa)*	text (date_dmy)
568	antiagregacion_fin<br>Show the field ONLY if: [antiagregacion] = 1	Fecha fin	radio<br>1 Fecha fin<br>2 Continúa en tratamiento
569	antiagregacion_ffin<br>Show the field ONLY if: [antiagregacion_fin] = '1'	Fecha fin<br>*(dd/mm/aaaa)*	text (date_dmy)
570	antiagregacion_seg<br>Show the field ONLY if: [antiagregacion] = 1	Perdido en el seguimiento	radio<br>1 Si<br>0 No

			Custom alignment: RH
571	antiagregacion_seg_fecha<br>Show the field ONLY if: [antiagregacion_seg] = 1	Fecha<br>*(dd/mm/aaaa)*	text (date_dmy)
572	antiagregacion_dias<br>Show the field ONLY if: [antiagregacion] = '1'	Total de días<br>*días*	text (integer)
573	otros_tto	¿Ha precisado otros tratamientos?<br>ej: inmunosupresores, anticoagulación, transfusiones, plasmaféresis	radio<br>1 Si<br>0 No<br>Custom alignment: RH
574	anti_tnf<br>Show the field ONLY if: [otros_tto] = '1'	Anti-TNF	radio<br>1 Si<br>0 No<br>Custom alignment: RH
575	anti_tnf_tipo<br>Show the field ONLY if: [anti_tnf] = 1		radio<br>1 Infliximab<br>2 Etanercept<br>3 Otro (especificar)<br>Custom alignment: LV
576	anti_tnf_tipo_otro<br>Show the field ONLY if: [anti_tnf_tipo] = 3	Especificar	text
577	anti_tnf_fecha<br>Show the field ONLY if: [anti_tnf] = '1'	Fecha<br>*(dd/mm/aaaa)*	text (date_dmy)
578	anti_tnf_dosis<br>Show the field ONLY if: [anti_tnf] = '1'	Dosis<br>*mg/kg/dosis*	text (number)
579	anti_tnf_dias<br>Show the field ONLY if: [anti_tnf] = '1'	Número de dosis<br>*dosis*	text (integer)
580	ciclosporina<br>Show the field ONLY if: [otros_tto] = '1'	Ciclosporina	radio<br>1 Si<br>0 No<br>Custom alignment: RH
581	ciclosporina_fecha<br>Show the field ONLY if: [ciclosporina] = '1'	Fecha<br>*(dd/mm/aaaa)*	text (date_dmy)
582	ciclosporina_dosis<br>Show the field ONLY if: [ciclosporina] = '1'	Dosis inicial<br>*mg/kg/dosis*	text (number)
583	ciclosporina_dosis_mant<br>Show the field ONLY if: [ciclosporina] = '1'	Dosis de mantenimiento<br>*mg/kg/dosis*	text (number)
584	ciclosporina_dias<br>Show the field ONLY if: [ciclosporina] = '1'	Número de días<br>*días*	text (integer)
585	ciclofosfamida<br>Show the field ONLY if: [otros_tto] = '1'	Ciclofosfamida	radio<br>1 Si<br>0 No<br>Custom alignment: RH
586	ciclofosfamida_fecha<br>Show the field ONLY if: [ciclofosfamida] = '1'	Fecha<br>*(dd/mm/aaaa)*	text (date_dmy)
587	ciclofosfamida_dosis<br>Show the field ONLY if: [ciclofosfamida] = '1'	Dosis<br>*mg/kg/día*	text (number)
588	ciclofosfamida_dias<br>Show the field ONLY if: [ciclofosfamida] = '1'	Número de días<br>*días*	text (integer)
589	anakinra<br>Show the field ONLY if: [otros_tto] = '1'	Anakinra	radio<br>1 Si<br>0 No

			Custom alignment: RH
590	anakinra_fecha<br>Show the field ONLY if: [anakinra] = '1'	Fecha<br>*(dd/mm/aaaa)*	text (date_dmy)
591	anakinra_dosis<br>Show the field ONLY if: [anakinra] = '1'	Dosis<br>*mg/kg/día*	text (number)
592	anakinra_dias<br>Show the field ONLY if: [anakinra] = '1'	Número de días<br>*días*	text (integer)
593	plamaferesis<br>Show the field ONLY if: [otros_tto] = '1'	Plasmaferesis	radio<br>1 Si<br>0 No<br>Custom alignment: RH
594	plasmaferesis_fecha<br>Show the field ONLY if: [plamaferesis] = '1'	Fecha<br>*(dd/mm/aaaa)*	text (date_dmy)
595	plasmaferesis_sesiones<br>Show the field ONLY if: [plamaferesis] = '1'	Número de sesiones<br>*sesiones*	text (integer)
596	plasmaferesis_volumen<br>Show the field ONLY if: [plamaferesis] = '1'	Volumen por sesión<br>*ml*	text (number)
597	anticoagulacion<br>Show the field ONLY if: [otros_tto] = '1'	¿Ha precisado anticoagulación?	radio<br>1 Si<br>0 No<br>Custom alignment: RH
598	anticoagulacion_heparinas<br>Show the field ONLY if: [anticoagulacion] = 1	Heparinas de bajo peso molecular	radio<br>1 Si<br>0 No<br>Custom alignment: RH
599	anticoagulacion_finicio_heparinas<br>Show the field ONLY if: [anticoagulacion_heparinas] = '1'	Fecha inicio<br>*(dd/mm/aaaa)*	text (date_dmy)
600	anticoagulacion_heparinas_fin<br>Show the field ONLY if: [anticoagulacion_heparinas] = 1	Fecha fin	radio<br>1 Fecha fin<br>2 Continúa en tratamiento
601	anticoagulacion_ffin_heparinas<br>Show the field ONLY if: [anticoagulacion_heparinas_fin] = '1'	Fecha fin<br>*(dd/mm/aaaa)*	text (date_dmy)
602	anticoagulacion_sintrom<br>Show the field ONLY if: [anticoagulacion] = 1	Sintrom	radio<br>1 Si<br>0 No<br>Custom alignment: RH
603	anticoagulacion_finicio_sintrom<br>Show the field ONLY if: [anticoagulacion_sintrom] = '1'	Fecha inicio<br>*(dd/mm/aaaa)*	text (date_dmy)
604	anticoagulacion_sintrom_fin<br>Show the field ONLY if: [anticoagulacion_sintrom] = 1	Fecha fin	radio<br>1 Fecha fin<br>2 Continúa en tratamiento
605	anticoagulacion_ffin_sintrom<br>Show the field ONLY if: [anticoagulacion_sintrom_fin] = '1'	Fecha fin<br>*(dd/mm/aaaa)*	text (date_dmy)
606	anticoagulacion_sintrom_seg<br>Show the field ONLY if: [anticoagulacion_sintrom] = 1	Perdido en el seguimiento	radio<br>1 Si<br>0 No<br>Custom alignment: RH
607	anticoagulacion_sintrom_seg_fecha<br>Show the field ONLY if: [anti	Fecha<br>*(dd/mm/aaaa)*	text (date_dmy)

	coagulacion_sintrom_seg] = 1		
608	anticoagulacion_otros<br>Show the field ONLY if: [anticoagulacion] = 1	Otros anticoagulantes	radio<br>1 Si<br>0 No<br>Custom alignment: RH
609	anticoagulacion_otros_espe<br>Show the field ONLY if: [anticoagulacion_otros] = '1'	Especificar	text
610	anticoagulacion_finicio_otros<br>Show the field ONLY if: [anticoagulacion_otros] = '1'	Fecha inicio<br>*(dd/mm/aaaa)*	text (date_dmy)
611	anticoagulacion_otros_fin<br>Show the field ONLY if: [anticoagulacion_otros] = 1	Fecha fin	radio<br>1 Fecha fin<br>2 Continúa en tratamiento
612	anticoagulacion_ffin_otros<br>Show the field ONLY if: [anticoagulacion_otros_fin] = '1'	Fecha fin<br>*(dd/mm/aaaa)*	text (date_dmy)
613	anticoagulacion_otros_seg<br>Show the field ONLY if: [anticoagulacion_otros] = 1	Perdido en el seguimiento	radio<br>1 Si<br>0 No<br>Custom alignment: RH
614	anticoagulacion_otros_seg_fecha<br>Show the field ONLY if: [anticoagulacion_otros_seg] = 1	Fecha<br>*(dd/mm/aaaa)*	text (date_dmy)
615	abciximab<br>Show the field ONLY if: [otros_tto] = '1'	¿Ha precisado Abciximab?	radio<br>1 Si<br>0 No<br>Custom alignment: RH
616	abciximab_fecha<br>Show the field ONLY if: [abciximab] = '1'	Fecha inicio<br>*(dd/mm/aaaa)*	text (date_dmy)
617	abciximab_dosis<br>Show the field ONLY if: [abciximab] = 1	Dosis carga<br>*mg/kg*	text (number)
618	abciximab_dosis_mant<br>Show the field ONLY if: [abciximab] = 1	Dosis mantenimiento<br>*mcg/kg/min*	text (number)
619	abciximab_horas<br>Show the field ONLY if: [abciximab] = 1	Numero de horas<br>*horas*	text (number)
620	transfusion<br>Show the field ONLY if: [otros_tto] = '1'	¿Ha precisado alguna transfusión?	radio<br>1 Si<br>0 No<br>Custom alignment: RH
621	transfusion_tipo<br>Show the field ONLY if: [transfusion] = '1'	Tipo	text
622	transfusion_fecha<br>Show the field ONLY if: [transfusion] = '1'	Fecha<br>*(dd/mm/aaaa)*	text (date_dmy)
623	comentarios_8	Comentarios	notes<br>Custom alignment: LV
624	tratamiento_complete	Complete?	dropdown<br>0 Incomplete<br>1 Unverified<br>2 Complete
Instrument: **Evolución y convalecencia**			
625	dias_fiebre	Días transcurridos desde el inicio del cuadro hasta el control definitivo de la fiebre<br>*días*	text (integer)
626	reagudizaciones	¿Presentó en paciente reagudizaciones?	radio<br>1 Si

			0 No<br><br>Custom alignment: RH
627	reagudizaciones_fecha<br>Show the field ONLY if: [rea gudizaciones] = '1'	Fecha<br>*(dd/mm/aaaa)*	text (date_dmy)
628	dias_normalizacion	Dias desde inicio del cuadro hasta normalización de la PCR<br>*días*	text (integer)
629	fecha_normalizacion	Fecha de normalización de la VSG<br>(anotar la fecha de la primera analítica con VSG normal)<br>*(dd/mm/aaaa)*	text (date_dmy)
630	fecha_normalizacion_plaque tas	Fecha de normalización de las plaquetas<br>(anotar la fecha de la primera analítica con plaquetas normales)<br>*(dd/mm/aaaa)*	text (date_dmy)
631	fecha_alta	Fecha de alta hospitalaria<br>*(dd/mm/aaaa)*	text (date_dmy)
632	reingreso	¿Ha precisado reingreso por patología relacionada con la EK?	radio<br>1 Si<br>0 No<br><br>Custom alignment: RH
633	reingreso_fecha<br>Show the field ONLY if: [rein greso] = '1'	Fecha<br>*(dd/mm/aaaa)*	text (date_dmy)
634	reingreso_motivo<br>Show the field ONLY if: [rein greso] = '1'	Especificar motivo de reingreso	notes<br>Custom alignment: LV
635	situacion_actual	Situación actual del paciente	radio<br>1 Alta médica<br>2 Perdido para el seguimiento<br>3 En seguimiento<br><br>Custom alignment: LV
636	fecha_alta_pediatria<br>Show the field ONLY if: [situ acion_actual] = '1' or [situaci on_actual] = '2'	Fecha<br>*(dd/mm/aaaa)*	text (date_dmy)
637	fecha_ult_contacto	Fecha del último contacto con el paciente<br>*(dd/mm/aaaa)*	text (date_dmy)
638	ingreso_ucip	¿Ha precisado ingreso en UCIP?	radio<br>1 Si<br>0 No<br><br>Custom alignment: RH
639	ingreso_ucip_motivo<br>Show the field ONLY if: [ingr eso_ucip] = '1'	Motivo	radio<br>1 Sospecha síndrome hemofagocítico<br>2 Miocarditis/disfunción cardiaca<br>3 Alteración del ritmo cardiaco<br>4 Sospecha de sepsis/shock séptico<br>5 Otros (especificar)<br><br>Custom alignment: LV
640	ingreso_ucip_motivo_otro<br>Show the field ONLY if: [ingr eso_ucip_motivo] = '5'	Especificar	text
641	ingreso_ucip_dias<br>Show the field ONLY if: [ingr eso_ucip] = '1'	Número de días en UCIP<br>*días*	text (integer)
642	sindrome_hemofagocitico	¿Ha cumplido criterios de síndrome hemofagocítico?<br>(según las guías HLH 2004. Pediatr Blood Cancer. 2007 Feb;48(2):124-31.	radio<br>1 Si<br>0 No<br><br>Custom alignment: RH
643	secuela_permanente	¿Presenta el paciente alguna secuela permanente?<br>(Indique si hay secuelas permanentes relacionadas con la enfermedad de Kawasaki o sus complicaciones)	radio<br>1 Si<br>0 No<br><br>Custom alignment: RH
644		Especificar cual	

	secuela_permanente_espe<br>Show the field ONLY if: [secuela_permanente] = '1'		notes<br>Custom alignment: LV
645	medicacion_cronica	¿Precisa el paciente medicación crónica relacionada con la enfermedad?	radio<br>1 Si<br>0 No<br>Custom alignment: RH
646	medicacion_cronica_espe<br>Show the field ONLY if: [medicacion_cronica] = '1'	Especificar cual	notes<br>Custom alignment: LV
647	fallecido	¿Ha fallecido el paciente como consecuencia de la enfermedad de Kawasaki?	radio<br>1 Si<br>0 No<br>Custom alignment: RH
648	fallecido_causa<br>Show the field ONLY if: [fallecido] = '1'	Causa de la muerte	notes<br>Custom alignment: LV
649	fecha_muerte<br>Show the field ONLY if: [fallecido] = '1'	Fecha de la muerte<br>*(dd/mm/aaaa)*	text (date_dmy)
650	comentarios_9	Comentarios	notes<br>Custom alignment: LV
651	evolucin_y_convalecencia_complete	Complete?	dropdown<br>0 Incomplete<br>1 Unverified<br>2 Complete
Instrument: **Scores (Autocalculados)**			
652	dias_enfermedad	Días de enfermedad<br>*días*	calc<br>Calculation: round(datediff([fecha_diagnostico], [fecha_alta_pediatria],"d","dmy"), 0)
653	edad	Edad<br>*meses*	calc<br>Calculation: round(datediff([fecha_diagnostico], [fecha_nac],"M","dmy"), 1)
654	egami_score	EGAMI - Score	calc<br>Calculation: if ([dias_enfermedad] <> "NaN" and [dias_enfermedad] <= 4, 1 , 0) + if ([alt] <> "NaN" and [alt] > 100, 1 , 0) + if ([plaquetas] <> "NaN" and [plaquetas] <= 300, 1 , 0) + if ([pcr] <> "NaN" and [pcr] >= 8, 1 , 0) + if ([edad] <> "NaN" and [edad] <= 6, 2 , 0)
655	dias_enfermedad2	Días de enfermedad<br>*días*	calc<br>Calculation: round(datediff([fecha_diagnostico], [fecha_alta_pediatria],"d","dmy"), 0)
656	edad2	Edad<br>*meses*	calc<br>Calculation: round(datediff([fecha_diagnostico], [fecha_nac],"M","dmy"), 1)
657	kobayashi_score	Kobayashi - Score	calc<br>Calculation: if ([dias_enfermedad] <> "NaN" and [dias_enfermedad] <= 4, 2 , 0) + if ([edad] <> "NaN" and [edad] <= 12, 1 , 0) + if ([alt] <> "NaN" and [alt] > 100, 1 , 0) + if ([plaquetas] <> "NaN" and [plaquetas] < 300, 1 , 0) + if ([pcr] <> "NaN" and [pcr] >= 10, 1 , 0) + if ([sodio] <> "NaN" and [sodio] <= 133, 2 , 0) + if ([neutrofilos_porcentaje] <> "NaN" and [neutrofilos_porcentaje] >= 80, 2 , 0)
658	sano_score	Sano - Score	calc<br>Calculation: if ([bilirrubina] <> "NaN" and [bilirrubina] >= 0.9, 1 , 0) + if ([ast] <> "NaN" and [ast] >= 200, 1 , 0) + if ([pcr] <> "NaN" and [pcr] >= 7, 1 , 0)
659	comentarios_10	Comentarios	notes<br>Custom alignment: LV
660	scores_autocalculados_complete	Complete?	dropdown<br>0 Incomplete<br>1 Unverified<br>2 Complete
Instrument: **Revisado por administrador**			
661	revisado_administrador		checkbox<br>1 revisado_administrador___1 Revisado por administrador

662	comentarios_11	Comentarios	notes<br>Custom alignment: LV
663	revisado_por_administrador_complete	Complete?	dropdown<br>0 Incomplete<br>1 Unverified<br>2 Complete
